# Morphological and molecular (28S rRNA) data of monogeneans (Platyhelminthes) infecting the gill lamellae of marine fishes in the Campeche Bank, southwest Gulf of Mexico

**DOI:** 10.3897/zookeys.783.26218

**Published:** 2018-09-11

**Authors:** Edgar F. Mendoza-Franco, Mariela del Carmen Rosado Tun, Allan de Jesús Duarte Anchevida, Rodolfo E. del Rio Rodríguez

**Affiliations:** 1 Instituto de Ecología, Pesquerías y Oceanografía del Golfo de México (EPOMEX), Avenida Héroe de Nacozari No. 480, CP. 24029, Universidad Autónoma de Campeche, San Francisco de Campeche, Campeche, México Universidad Autónoma de Campeche San Francisco de Campeche Mexico

**Keywords:** Ariidae, *
Choricotyle
*, *
Euryhaliotrema
*, Haemulidae, *
Haliotrematoides
*, *
Hamatopeduncularia
*, Lutjanidae, *
Microcotyle
*, *
Microcotyloides
*, Monogenea, *
Neotetraonchus
*, Sparidae

## Abstract

During the examination of 913 fish specimens belonging to four families in the Campeche Bank (Gulf of Mexico), 23 gill ectoparasitic monogenean species were found, which belong to three families: Dactylogyridae, Microcotylidae and Diclidophoridae. The species *Euryhaliotremaamydrum*, *E.carbuncularium*, *E.dunlapae*, *E.fajeravilae*, *E.fastigatum*, *E.longibaculum*, *E.paracanthi*, *E.tubocirrus*, *Haliotrematoidescornigerum*, *H.gracilihamus*, *H.heteracantha*, *H.longihamus*, *H.magnigastrohamus*, *H.striatohamus*, *Hamatopedunculariabagre*, *Neotetraonchusbravohollisae*, and *N.felis* (all Dactylogyridae) were found on the hosts *Lutjanussynagris*, *L.griseus*, *Ariopsisfelis*, *Bagremarinus*, *Archosargusrhomboidalis*, and *Haemulonplumieri*. Additionally, *Microcotylearchosargi*, *Microcotyle* sp., and *Microcotyloidesincisa* (all Microcotylidae) were found on *L.griseus* and *A.rhomboidalis*; finally, *Choricotyle* sp. 1, *Choricotyle* sp. 2, and *Choricotyle* sp. 3 (all Diclidophoridae) were found on *H.plumieri*. The prevalence, abundance, mean intensity of infection, and supplementary taxonomic revisions for all monogeneans found are provided. Partial sequences of the 28S rRNA gene were also obtained for monogeneans of ariid, sparid, and haemulid host fishes to explore their systematic position within the Monogenea. New locality and host records for some previously described species of *Euryhaliotrema*, *Hamatopeduncularia*, *Microcotyle*, and *Choricotyle* from lutjanid, ariid, sparid, and haemulid hosts were reported. The present study adds evidence supporting the interoceanic occurrence of the same monogenean species (on lutjanids) on the west-east Atlantic and Pacific Oceans (= amphiamerican species). As previously suggested, there are at least, two possibilities to explain that parasite distribution: differentiation of morphological features in these monogeneans have resulted in only slight to insignificant morphological changes developing over the extended period of 3.2 mya (when the Isthmus of Panama was closing) and/or speciation is only evident at molecular level.

## Introduction

The Campeche Bank (southwest Gulf of Mexico) represents an important marine ecosystem characterized by a high biodiversity, which is threatened by important overfishing and energy (petroleum) extraction activities (Soto et al. 2014). For example, oil can affect marine wildlife by physical effects, i.e., death by suffocation, with oil blocking air passageways or fish gills (NOAA 2018, Overstreet and Hawkins 2017). Because of its economic impact on Mexico´s economy, the Campeche Bank is considered a strategic region in the national plans for the social and economic development of Mexico (Piñeiro 2001).The knowledge of the diversity, abundance and distribution of species is the base for developing management plans for threatened species and preserving its natural resources for ecological and economic purposes (Ocean Conservancy 2011).

Biodiversity is widely considered to correlate with ecosystem health, the presence or abundance of parasites becomes part of that positive biodiversity. Otherwise, the fewer the parasites observed, the worse the environmental conditions and thus the biodiversity (see Vidal-Martínez and Wunderlich 2017). Therefore, parasite biodiversity information can be critical for the control and safe management of commercial fish species (Vignon and Sasal 2010, Quiazon 2015). However, parasites remain an underestimated component of the total biodiversity in many regions (Lafferty et al. 2015).

Despite their ecological and environmental effects, there have been few studies aimed at collecting and examining fish samples for parasites; in consequence, many parasite species go undetected or are poorly studied. Low availability and poor quality of material for examination also adds to this problem. This lack of knowledge about biodiversity also prevents us for understanding the connectivity between the northern and southern Gulf fisheries.

As part of a research project on fish parasite biodiversity in the Campeche Bank, we had the opportunity to undertake a survey of ectoparasitic monogeneans infecting the gill lamellae of six marine fish species. Here, we provided: 1) supplementary information and illustrations of the sclerotised and/or soft structures of the monogenean species found; 2) information on the prevalence and intensity of infections at each site sampled; and 3) data on the biometrical variability of individual monogenean species collected on different hosts. In addition, partial sequences of the 28S rRNA gene (D1–D3) were amplified from monogeneans of ariid, sparid, and haemulid hosts to explore their systematic position within the Monogenea. The occurrence of the monogenean species found with respect to the west-east Atlantic and Pacific divide is briefly discussed.

## Materials and methods

We studied the most abundant fish species (Diario Oficial de la Federación 2012), resulting in six species that were caught from three coastal locations in the state of Campeche [southwestern coast of the Gulf of Mexico: San Francisco (19°55.988'N; 90°41.969'W), Seyba Playa (19°42.580'N; 90°44.155'W), and Champoton (19°16.390'N, 90°49.194'W)]. Fish were collected over a period of eight months (from January to August 2015) using gill nets. Fish were kept on ice for a maximum of 12 hours before their gills were removed and placed in ﬁngers bowls containing a 4% formaldehyde solution to ﬁx ectoparasites. Parasites were subsequently detached from the gills using fine needles under a dissecting microscope, stained with Gomori’s trichrome stain and mounted in Canada balsam (Vidal-Martínez et al. 2001). A selection of specimens was mounted on slides using a mixture of lactic acid (LA) and glycerin-ammonium picrate (GAP) and then remounted in Canada balsam (Mendoza-Franco et al. 2013) to obtain measurements of the haptoral structures and copulatory complex.

All other measurements were obtained from unﬂattened specimens stained with Gomori’s trichrome stain. Measurements are in micrometers and expressed as the mean followed by the range and number (n) of structures measured in parentheses; body length and greatest width includes and exclude the haptor, respectively. Illustrations were prepared with the aid of a drawing tube on a Leica DM 2500 microscope with differential interference contrast and phase contrast optics. The direction of the coil (clockwise vs. counterclockwise) of the copulatory organ was determined following Kritsky et al. (1985). Reference specimens were deposited in the Colección Nacional de Helmintos, Universidad Nacional Autónoma de México, Mexico City, Mexico (CNHE). In addition, the following museum specimens were examined: voucher, *Euryhaliotrematubocirrus* (Zhukov, 1976) Kritsky & Boeger, 2002 (CNHE 10222); voucher, *Euryhaliotremalongibaculum* (Zhukov, 1976) Kritsky & Boeger, 2002 (CNHE 10221); voucher, *Haliotrematoidescornigerum* (Zhukov, 1976) Kritsky, Yang & Sun, 2009 (CNHE 10217); voucher, *Haliotrematoidesmagnigastrohamus* (Zhukov, 1976) Kritsky, Yang & Sun, 2009 (CNHE 10220); voucher, *Euryhaliotrematorquecirrus* (Zhukov, 1976) Kritsky & Boeger, 2002 (CNHE 10223); voucher, *Haliotrematoideslongihamus* (Zhukov, 1976) Kritsky, Yang & Sun, 2009 (CNHE 10219); voucher, *Haliotrematoidesheteracantha* (Zhukov, 1976) Kritsky, Yang & Sun, 2009 (CNHE 10218); 5 vouchers, *Paramicrocotyletampicensis* Caballero y Caballero & Bravo-Hollis, 1972 (CNHE 226-10); paratype, *Paramicrocotyleatriobursata* Caballero y Caballero & Bravo-Hollis, 1972 (CNHE 266-9). Host body lengths were expressed as total length (TL) in cm.

Prior to DNA analysis, parasites were fixed with 96% ethanol and individually identified based on the morphology of their haptors. The haptor of each specimen was removed using syringe needles (used for insulin injections) and mounted unstained in a mixture of LA and GAP. The body of the worm was transferred to a labeled Eppendorf tube containing 96 % ethanol and stored at room temperature until required for molecular evaluation. Processed haptors were then remounted in Canada balsam (see Mendoza-Franco et al. 2009) and studied using an immersion oil objective on a DM2500 Leica microscope. These haptors were kept as molecular vouchers (hologenophore, i.e., the voucher specimen from which the molecular sample was obtained; see Astrin et al. 2013) and deposited in the CNHE.

Two to ten bodies of excised specimens from the gills of ariid, sparid, and haemulid fishes collected at each of the three sampling sites were placed individually in a 0.2 µl Eppendorf tube for genomic DNA extraction. Genomic DNA of each individual was extracted using 20 µl Chelex (100 sodium) and 5 µl proteinase K (at 10mg µl^-1^) to lyse parasite tissues. Specimens were immediately incubated for 3 h and 15 minutes at 96 °C to denature the proteinase K. Volumes of 5 µl were taken from each lysed preparation to serve as template DNA samples in the PCR assays. A fragment of the 28S rRNA gene (D1–D3) was amplified using the polymerase chain reaction (PCR). The internal primers Halio-F (5´-ACCCGCTGAATTTAAGCAT-3´) and Halio-R (5´-TGGTCCGTGTTTCAAGAC-3´) were used for amplification (García-Vásquez et al. 2015). All PCR reactions were performed in a final volume of 50 μl composed of 5 μl 10× PCR buffer, 1.5 μl 10 mM dNTPs mixture (10 μM each), 4.0 μl 2.0mM MgCl_2_, 1.5μl of each primer (10 μM), 5 μl template DNA, 0.24 μl Taq DNA polymerase (1.2 units), and 31.26 μl of sterile distilled water. The following thermocycling profile was used: initial denaturation at 94 °C for 2 min, followed by 35 cycles of 94 °C for 30 sec, annealing at 55 °C for 30 sec and final extension at 72 °C for 3 min. The mounts or permanent preparations containing a haptor used to identify parasite specimens for which the body was used to amplify DNA were deposited in the CNHE.

### Alignment, phylogenetic analyses, and sequence divergence

28S (D1–D3) sequences obtained in the current study were aligned with that of other monogenean species available in GenBank using Muscle algorithm implemented in Mega 7 (Kumar et al. 2015) and adjusted manually with the program Mesquite 2.75 (Maddison and Maddison 2011). The software jModelTest version 2.1.10 (Darriba et al. 2012) was used to select the best model of evolution for our dataset. The model (GTR+I+G) was selected based on the Akaike information criteria. Maximum likelihood (ML; 1000 Bootstrap) and Bayesian Inference (BI) analyses were performed using Mega 7 and Mr. Bayes version 3.2, respectively (Huelsenbeck and Ronquist 2001). Mr. Bayes was used based on Markov chains model with burning periods every 1,000 generations to reach a consensus after 400,000 generations. Numbers at the interior branches of the consensus tree represent posterior probabilities (PP) and booptstrap of maximum likehoods. Trees were drawn using the program Fig. Tree V.1.4.3 (Drummond et al. 2006). The genetic divergence among species [*Hamatopedunculariabagre* Hargis, 1955, *Microcotylearchosargi* MacCallum, 1913, *Haliotrematoidesstriatohamus* (Zhukov, 1981) Mendoza-Franco, Reyes-Lizama & González-Solís, 2009, and *Choricotyle* spp.] was estimated using the uncorrected “p-distances” method with the program MEGA v. 5 (Tamura et al. 2011).

## Results

A total of 913 fish specimens across six species [*Lutjanussynagris* (Linnaeus, 1758), *Lutjanusgriseus* (Linnaeus, 1758) (Lutjanidae); *Ariopsisfelis* (Linnaeus, 1766); *Bagremarinus* (Mitchill, 1815) (Ariidae); *Archosargusrhomboidalis* (Linnaeus, 1758) (Sparidae); and *Haemulonplumieri* (Lacepède, 1801) (Haemulidae)] were collected at the three locations aforementioned (San Francisco: 308 individuals–33.7%; Champoton: 335–36.7% and Seyba: 270–29.6%). 803 of these specimens (88%) were infected with monogeneans. A total of 23 monogenean species from three families was found (see Table 1).

**Table 1.** Gill ectoparasitic monogeneans (Platyhelminthes) on marine fishes from the Campeche Bank (southwest Gulf of Mexico).

Parasite family/species	Host families and species					
	Lutjanidae		Ariidae		Sparidae	Haemulidae
	* Lutjanus synagris *	* Lutjanus griseus *	* Ariopsis felis *	* Bagre marinus *	* Archosargus rhomboidalis *	* Haemulon plumieri *
*** Dactylogyridae ***						
* Euryhaliotrema amydrum *	–	–	–	–	+⁰	–
* E. carbuncularium * ^‡^	–	–	–	–	+⁰	–
* E. dunlapae *	–	–	–		+⁰	–
* E. fajeravilae * ^†^	–	+⁰	–	–	–	–
* E. fastigatum * ^†^	–	+/	–	–	–	–
* E. longibaculum *	+/	+⁰	–	–	–	–
* E. paracanthi * ^†^	–	+/	–	–	–	–
* E. tubocirrus *	+/	+/	–	–	–	–
* Haliotrematoides cornigerum *	+/	–	–	–	–	–
* Hal. gracilihamus *	–	+/	–	–	–	–
* Hal. heteracantha * ^†^	+/	+/	–	–	–	–
* Hal. longimanus *	+/	–	–	–	–	–
* Hal. magnigastrohamus *	+/	–	–	–	–	–
* Hal. striatohamus * ^‡^	–	–	–	–	–	+/
* Hamatopeduncularia bagre * ^‡^	–	–	–	+/	–	–
* Neotetraonchus bravohollisae *	–	–	+/	–	–	–
* N. felis * ^‡^	–	–	+/	–	–	–
*** Microcotylidae ***						
* Microcotyle archosargi * ^‡^	–	–	–	–	+⁰	–
*Microcotyle* sp.	–	–	–	–	+/	–
* Microcotyloides incisa * ^†^	–	+/	–	–	–	–
*** Diclidophoridae ***						
*Choricotyle* sp. 1^‡^	–	–	–	–	–	+⁰
*Choricotyle* sp. 2	–	–	–	–	–	+⁰
*Choricotyle* sp. 3	–	–	–	–	–	+⁰

^†^ = Occurring on the Pacific and Atlantic sides of North America; ^‡^ = Sequenced in the present study; ⁰ = New host and locality records; / = New locality record.

### Dactylogyridae Bychowsky, 1933

#### Euryhaliotrema

Taxon classificationAnimaliaMazocraeideaDactylogyridae

Kritsky & Boeger, 2002

Euryhaliotrema Kritsky & Boeger, 2002: 12, fig. 1; Kritsky 2012: 230 (revised and amended).
Euryhaliotrema amydrum Kritsky & Bakenhaster, 2011: 64, figs 17–24.

##### Type host.

*Archosargusprobatocephalus* (Walbaum, 1792)

##### Present study.

*A.rhomboidalis* (new host)

##### Locality/prevalence, mean abundance and intensity range.

**San Francisco**: 18 fish (mean TL 26.9 cm; range 17–23.6) infected of 18 examined (100 %); abundance, 37; intensity of infection, 22–51 worms. **Seyba Playa**: 25 fish (TL 28.4; 19.2–30.5) infected of 25 examined (100 %); abundance, 37; intensity of infection, 29–47. **Champoton**: 45 fish (TL 28.2; 24.7–30.5) infected of 45 examined (100 %); abundance, 23; intensity of infection, 8–35.

##### Supplementary observations

**(measurements based on six specimens).** Body 336 (305–350; 6) long; greatest width 80 (70–90; 4). Pharynx 23 wide. MCO a counterclockwise coil of 1–2 rings, proximal ring 11 (10–12; 5) diameter. Haptor 77 (67–84; 4) wide. Ventral anchor 31 (28–36; 10) long; dorsal anchor 43 (40–45; 9) long. Ventral bar 35 (30–41; 7) long; dorsal bar 33 (32–34; 4) long.

##### Comments.

This species was originally described on the sheepshead *A.probatocephalus* from the Indian River Lagoon in Florida (Kritsky and Bakenhaster 2011). This species is mainly characterized in having a tightly coiled MCO and dorsal anchor roots approaching the length of the dorsal anchor shaft. Differences in the length of the dorsal anchors between present specimens and those of *E.amydrum* originally described were found (i.e. length 40–45 vs. 49 53), but the worms are clearly conspecific. Montoya-Mendoza et al. (2015) reported *E.amydrum* on *A.probatocephalus* from Alvarado Lagoon and El Conchal estuary in Veracruz (Gulf of Mexico). However, these latter authors did not provided any accession number for their parasite specimens apparently deposited in the CNHE. Then, we could not corroborate Montoya´s finding.

##### Specimens deposited.

Six reference specimens in the CNHE (10607).

#### Euryhaliotrema carbuncularium

Taxon classificationAnimaliaMazocraeideaDactylogyridae

Kritsky & Bakenhaster, 2011

##### Type host.

*Archosargus probatocephalus*

##### Present study.

*A.rhomboidalis* (new host)

##### Supplementary observations

**(measurements based on three specimens).** Ventral anchor 48 (46–49; 4) long; dorsal anchor 41 (48–54; 3) long. Haptor 60 wide. Ventral bar 32–34 long.

##### Comments.

In *A.rhomboidalis*, a simultaneous infection with *E.amydrum* was found. Since all worms could not be identified, the data on infection rate relate to *E.carbuncularium* and *E.amydrum*. *Euryhaliotremacarbuncularium* was originally described on *A.probatocephalus* from the Indian River Lagoon near Malabar, Brevard County, Florida (Kritsky and Bakenhaster 2011).

##### Molecular data.

**Figure 1.**
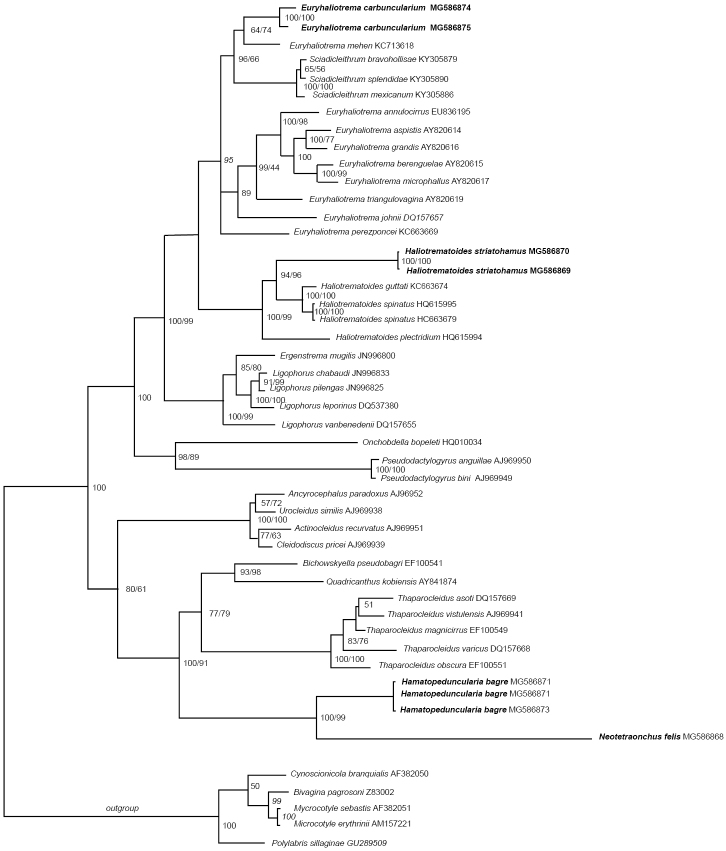
Molecular phylogeny of the Dactylogyridae estimated by methods of Bayesian inference (BI) and maximum likelihood (ML) using partial sequences of the 28S rRNA gene (D1–D3). Species newly sequenced for this study are in bold. Species belonging to Microcotylidae were used as outgroups. The species name is followed by the GenBank sequence ID. Posterior probabilities of the BI followed by ML are given above the branches.

The present study also provided the ﬁrst molecular data of *E.carbuncularium*; there are two sequences (676 and 856 bp, respectively) of individual specimens of this monogenean species included within the analyses that shows that this species forms a sister lineage to that containing *Euryhaliotremamehen* (Soler-Jiménez, García-Gasca & Fajer-Ávila, 2012) Kritsky, 2012, which is known on *Lutjanusguttatus* (Steindachner, 1869) in the Eastern Pacific (see Figure 1).

##### Specimens deposited.

Three reference specimens in the CNHE (10608).

Two slides, each containing a haptor of a specimen of *E.carbuncularium* used to amplify its DNA are deposited in the CNHE (10622).

##### Representative DNA sequence.

GenBank accession number MG586874, MG586875.

#### Euryhaliotrema dunlapae

Taxon classificationAnimaliaMazocraeideaDactylogyridae

Kritsky & Bakenhaster, 2011

##### Type host.

*Archosargus probatocephalus*

##### Present study.

*A.rhomboidalis* (new host)

##### Supplementary observations

**(measurements based on seven specimens).** Body 285 (200–360; 7) long; greatest width 63 (60–73; 3). MCO 17 (14–21; 7) long; proximal ring of the MCO 9 (8–10; 4) diameter. Haptor 54 (45–60; 4) wide. Ventral anchor 30 (29–31; 7) long; dorsal anchor 41 (37–47; 6) long. Hook pair 1, 14 (3) long.

##### Comments.

A simultaneous infection with *E.amydrum* and *E.carbuncularium* occurred on *A.rhomboidalis*. Since all worms could not be identified, the data on infection rate relate to *E.dunlapae*, *E.amydrum*, and *E.carbuncularium*. *Euryhaliotremadunlapae* was originally described on *A.probatocephalus* from the Indian River Lagoon near Malabar, Brevard County, Florida (Kritsky and Bakenhaster 2011). Morphometrical comparison between present specimens and those originally described did not reveal a significant difference. As mentioned above for *E.amydrum*, Montoya-Mendoza et al. (2015) also reported *E.dunlapae* on *A.probatocephalus* in Veracruz, Mexico; however, they did not provide any accession number for these specimens of *E.dunlapae*.

##### Specimens deposited.

Seven reference specimens in the CNHE (10609).

#### Euryhaliotrema fajeravilae

Taxon classificationAnimaliaMazocraeideaDactylogyridae

Kritsky & Mendoza-Franco, 2012

##### Type host.

*Lutjanusargentiventris* (Peters, 1869)

##### Present study.

*L.griseus* (new host)

##### Locality/prevalence, mean abundance and intensity range.

**San Francisco**: two fish (mean TL 28.1cm; range 21.6–39) infected of 65 examined (3 %); abundance, 0.1; intensity of infection, 1–2 worms. **Champoton**: 1 fish (TL 28.3; 25.2–37) infected of 37 examined (2.7 %), abundance, 0.05; intensity of infection, 2.

##### Supplementary observations

**(measurements based on four specimens).** Body 327 (200–380; 4) long; greatest width 76–80. Haptor 70 (65–75; 3) wide. MCO 19 (18–21; 4) long. Ventral anchor 39 (35–44; 8) long; dorsal anchor 55 (47–59; 8) long. Ventral bar 40 (33–54; 4) long; dorsal bar 37 (30–46; n = 4) long.

##### Comments.

This species was described from *L.argentiventris* from the Perlas Archipielago, Panama by Kritsky and Mendoza-Franco (in Kritsky 2012). *Euryhaliotremafajeravilae* is distinguished from other species of the genus infecting lutjanids by having larger anchors and a noticeably smaller copulatory complex. The morphometrics of the present specimens did not differ from that of the original description.

##### Specimens deposited.

Four reference specimens in the CNHE (10614).

#### Haliotrema fastigatum

Taxon classificationAnimaliaMazocraeideaDactylogyridae

Zhukov, 1976

Haliotrema fastigatum Zhukov, 1976: 43, fig. 10; Kritsky and Boeger 2002: 33 (transferred to Euryhaliotrema); Kritsky 2012: 237–239, figs 11–17 (redescribed).

##### Type host.

*Lutjanusapodus* (Walbaum, 1792)

##### Present study.

*L. griseus*

##### Locality/prevalence, mean abundance and intensity range.

**San Francisco**: 63 fish (mean TL 28.1cm; range 21.6–39) infected of 65 examined (97 %); abundance, 12; intensity of infection, 10–18 worms. **Seyba Playa**: 40 fish (TL 28.5; 23–37) infected of 45 examined (88.8 %); abundance, 8; intensity of infection, 2–11. **Champoton**: 27 fish (TL 28.3; 25.2–37) infected of 37 examined (73 %); abundance, 4; intensity of infection, 4–11.

##### Supplementary observations

**(measurements based on ten specimens).** Body 356 (300–460; 9) long; greatest width 72 (54–95; 8). Haptor 70 (47–85; 8) wide. Pharynx 16 (13–18; 8) wide. MCO 32 (28–34; 7) long. Proximal ring of the MCO 18 (12–20; 8) diameter. Accessory piece 23 (21–24; 3) long. Ventral anchor 29 (27–30; 10) long; dorsal anchor 41 (38–45; 6) long. Ventral bar 36 (34–40; 7) long; dorsal bar 37 (35–46; 5) long. Hook 12 (11–12; 7) long.

##### Comments.

Zhukov (1976) originally described this species as *Haliotremafastigatum* from *L.apodus* and *Lutjanusjocu* (Bloch & Schneider, 1801) from the area Havana (Gulf of Mexico). In 2002, Kritsky and Boeger transferred this species to *Euryhaliotrema* as *E.fastigatum* based on details presented in the original description (Zhukov 1976) of the copulatory complex, internal organs, and haptoral armament according with the diagnosis of *Euryhaliotrema*. Later, Kritsky (2012) redescribed *E.fastigatum* based on specimens collected from *L.griseus* and other lutjanids (*L.apodus*, *L.jocu*, and *L.argentiventris*) from Florida and off Taboga Island, Perlas Archipielago, Isla Saboga, and Isla Tabugilla (all from the Pacific of Panama). *Euryhaliotremafastigatum* is characterized in having a thinning of the base of the dorsal anchor near its junction with the anchor shaft and by lacking by lacking an articulation process in the copulatory complex. Measurements and the morphology of the sclerotized structures of the present specimens do not differ significantly from that figured in the redescription of *E.fastigatum*. This monogenean species has also been reported on *Lutjanusanalis* (Cuvier, 1828) and *L.griseus* from Puerto Rico and off Venezuela (Bosques-Rodriguez 2004, Fuentes Zambrano et al. 2003, Fuentes Zambrano and Silva Rojas 2006, Kritsky 2012).

##### Specimens deposited.

Ten reference specimens in the CNHE (10621).

#### Haliotrema longibaculum

Taxon classificationAnimaliaMazocraeideaDactylogyridae

Zhukov, 1976

Haliotrema longibaculum Zhukov, 1976: 39, fig. 6; Kritsky and Boeger 2002: 32 (transferred to Euryhaliotrema); Kritsky 2012: 242–244, figs 30–36 (redescribed).

##### Type host.

*Lutjanusmahogoni* (Cuvier, 1828)

##### Present study.

*L.synagris* and *L.griseus* (new host)

##### Locality/prevalence, mean abundance and intensity range on *L.synagris*.

**San Francisco**: 68 fish (mean TL 28.2 cm; range 20–35.7) infected of 70 examined (97%); abundance, 12; intensity of infection, 8–19 worms. **Seyba Playa**: 77 fish (TL 28.4; 19.2–30.5) infected of 79 examined (97.5 %); abundance, 14; intensity of infection, 6–27. **Champoton**: 70 fish (TL 28.2; 24.7–30.5) infected of 75 examined (93.3%); abundance, 12; intensity of infection, 4–18.

##### Supplementary observations

**(measurements based on ten specimens on *L.synagris*).** Body 217 (190–270; 8) long; greatest width 62 (50–72; 4). Haptor 65 (55–80; 8) wide. Pharynx 19 (15– 24; 6) wide. MCO 21 (18–26; 6) long. Ventral anchor 25 (24–26; 9) long; dorsal anchor 37 (32–40; 11) long. Ventral bar 43 (33–49; 8) long; dorsal bar 32 (30–33; 5) long.

##### Comments.

*Euryhaliotremalongibaculum* was originally described and depicted (as *Haliotremalongibaculum*) from *L.synagris* and *L.mahogoni* collected off Cuba (Area Havana) (Zhukov 1976, Kritsky 2012). Later, Kritsky and Boeger (2002) transferred the species to *Euryhaliotrema* based on Zhukov´s original description and drawings. The present specimens fit the diagnosis of *E.longibaculum*, which was redescribed by Kritsky (2012) based on specimens found in *L.synagris* from Florida, USA. *Euryhaliotremalongibaculum* is characterized by having dorsal anchors with an elongate superficial root, poorly developed deep root and elongate point extending anteriorly near to the level of the union of the anchor shaft and base, and an articulation process in the copulatory complex connecting the accessory piece to the base of the MCO. Morphometrical comparison of the present material with the redescription of this species provided by Kritsky (2012) did not reveal any differences. Recently, Montoya-Mendoza et al. (2016) reported *E.longibaculum* (voucher CNHE 10221), from *L.synagris* from Santiaguillo Reef, Veracruz (Gulf of Mexico). Examination of that voucher allowed us to confirm the species identity.

##### Specimens deposited.

Ten reference specimens in the CNHE (10601).

#### Haliotrema paracanthi

Taxon classificationAnimaliaMazocraeideaDactylogyridae

Zhukov, 1976

Haliotrema paracanthi Zhukov, 1976: 42–43, fig. 9; Kritsky and Boeger 2002: 32 (transferred to Euryhaliotrema); Kritsky 2012: 239–240, figs 18–23 (redescribed).

##### Type host.

*Lutjanus apodus*

##### Present study.

*L. griseus*

##### Locality/prevalence, mean abundance and intensity range.

**San Francisco**: 6 fish (mean TL 28.1cm; range 21.6–39) infected of 65 examined (9 %); abundance, 0.09; intensity of infection, 1 worm. **Seyba Playa**: 2 fish (TL 28.5; 23–37) infected of 45 examined (4 %); abundance, 0.06; intensity of infection, 1–2; **Champoton**: 1 fish (TL 28.3; 25.2–37) infected of 37 examined (2.7 %); abundance, 0.02; intensity of infection, 1.

##### Supplementary observations

**(measurements based on seven specimens).** Body 348 (295–445; 6) long; greatest width 70 (70–92; 3). Haptor 71 (62–82; 4) wide. MCO 28 (25–34; 8) long. Proximal ring of the MCO 17 (13–20; 7) diameter. Ventral anchor 24 (23–25; n = 12) long; dorsal anchor 24 (24–25; n = 11) long. Ventral bar 31 (27–38; 7) long; dorsal bar 23 (23–24; 4) long. Hook 13 (12–14; 8) long.

##### Comments.

This species was originally described as *Haliotremaparacanthi* by Zhukov (1976) from *L.apodus* from the Area Havana (off Cuba) and later transferred to *Euryhaliotrema* by Kritsky and Boeger (2002) based on the drawings presented in the original description of Zhukov (1976). Subsequently, Kritsky (2012) redescribed *E.paracanthi* based on specimens collected from *L.jocu* and other lutjanids (*L.argentiventris* and *L.griseus*) from Florida and off Taboga Island, and Perlas Archipielago (both from Panama). *Euryhaliotremaparacanthi* is differentiated from other species of *Euryhaliotrema* infecting lutjanids by possessing a subterminal spine or hook on the accessory piece. The morphometrics of the present specimens did not differ from those reported in the redescription of *E.paracanthi*.

##### Specimens deposited.

Seven reference specimens in the CNHE (10613).

#### Haliotrema tubocirrus

Taxon classificationAnimaliaMazocraeideaDactylogyridae

Zhukov, 1976

Haliotrema tubocirrus Zhukov, 1976: 40–41, fig. 7; Kritsky and Boeger 2002: 33 (transferred to Euryhaliotrema); Kritsky 2012: 234–237, figs 1–10 (redescribed).

##### Type host.

*Lutjanus synagris*

##### Present study.

*L.synagris* and *L.griseus*

##### Locality/prevalence, mean abundance and intensity range on *L.synagris*.

**San Francisco**: 69 fish (mean TL 28.2 cm; range 20–35.7) infected of 70 examined (98.6%); abundance, 14; intensity of infection, 8–20 worms. **Seyba Playa**: 77 fish (TL 28.4; 19.2–30.5) infected of 79 examined (97.5 %); abundance, 15; intensity of infection, 11–22. **Champoton**: 75 fish (TL 28.2; 24.7–30.5) infected of 75 examined (100 %); abundance, 17; intensity of infection, 5–27.

##### Supplementary observations

**(measurements based on twelve specimens on *L.synagris*).** Body 518 (402–640; 12) long; greatest width 81 (65–100; 8). Haptor 74 (53–100; 8) wide. Pharynx 28 (20–35; n = 6) wide. Male copulatory organ (MCO) 40 (35–43; n = 11) long. Accessory piece 29 (28–31; 5) long. Proximal ring of the MCO 24 (19–33; n = 8) diameter. Ventral anchor 26 (23–28; 16) long; dorsal anchor 26 (21–30; 13) long. Ventral bar 36 (32–42; 8) long; dorsal bar 28 (25–32; 8) long. Hook 13 (11–13; 12) long.

##### Comments.

This species was originally described as *Haliotrematubocirrus* from the gills of *L.synagris*, *L.analis*, *L.apodus*, *Lutjanuscyanopterus* (Cuvier, 1828) and *Rhomboplitesaurorubens* (Cuvier, 1829) from the environs of Havana, Cuba (Zhukov 1976). Kritsky and Boeger (2002) transferred this species to *Euryhaliotrema* based on the description and drawings provided in the original description by Zhukov (1976). Since then, *E.tubocirrus* has been reported on other lutjanids [*Lutjanusvivanus* (Cuvier, 1828), *L.griseus* and *Lutjanusbuccanella* (Cuvier, 1828), *L.mahogoni*] from Puerto Rico (see these reports in Kritsky 2012).

The present specimens were identified as *E.tubocirrus* according to the redescription of this species made by Kritsky (2012) based on specimens found on other five lutjanids: *Lutjanuscampechanus* (Poey, 1860), *L.griseus*, *L.jocu*, *L.synagris* and *R.aurorubens* from the northern Gulf of Mexico (Mississippi coast and Florida). *Euryhaliotrematubocirrus* is characterized by having anchors with moderately developed superficial root, short deep root, slightly arced shaft, elongate point, two complete rings in the MCO, and accessory piece multi-branched. Previous fish species reported as hosts of *E.tubocirrus* were collected off Cuba (Area Havana), Puerto Rico and Mississippi coast and Florida in USA. Recently, Montoya-Mendoza et al. (2016) reported *E.tubocirrus* (voucher CNHE 10222), from *L.synagris* from Santiaguillo Reef, Veracruz (Gulf of Mexico). Examination of that voucher allowed us to confirm the conspecificity of present specimens with those collected by Montoya-Mendoza et al. (2016).

##### Specimens deposited.

Twelve reference specimens in the CNHE (10604).

#### Haliotrema cornigerum

Taxon classificationAnimaliaMazocraeideaDactylogyridae

Zhukov, 1976

Haliotrema cornigerum Zhukov, 1976: 33–34, fig. 1; Kritsky et al. 2009b: 42 (transferred to Haliotrematoides).

##### Type host.

*Lutjanussynagris*.

##### Present study.

*L.synagris*.

##### Locality/prevalence, mean abundance and intensity range.

**San Francisco**: 35 fish (mean TL 28.2 cm; range 20–35.7) infected of 70 examined (50 %); abundance, 3; intensity of infection, 1–7 worms. **Seyba Playa**: 24 fish (TL 28.4; 19.2–30.5) infected of 79 examined (30 %); abundance, 1; intensity of infection, 2–4. **Champoton**: 48 fish (TL 28.2; 24.7–30.5) infected of 75 examined (64 %); abundance, 14; intensity of infection, 1–21.

##### Supplementary observations

**(measurements based on six specimens).** Body 422 (320–545; 6) long; greatest width 65 (3). Haptor 60 (52–65; 5) wide. Pharynx 20 (18–23; 4) wide. MCO 56 (49–60; 6) long. Ventral anchor 40 (38–42; 6) long; dorsal anchor 53 (51–56; 10) long. Ventral bar 34 (33–34; 4) long; dorsal bar 30 (28–33; 5) long.

##### Comments.

Identification of present specimens is based on diagnosis provided by Kritsky et al. (2009b) which transferred this species from *Haliotrema* to *Haliotrematoides* on the basis of study of Zhukov’s (1976) original figures and those provided by Bosques-Rodríguez (2004). This species is characterized in having an inner spur on the dorsal anchor shaft and shaft of MCO having a proximal loop (see Kritsky et al. 2009). *Haliotrematoidescornigerum* is currently reported from *L.synagris* and *L.mahogoni* from the Bay of Campeche (Area Havana) and Puerto Rico (Zhukov 1976, Bosques-Rodríguez 2004, Kritsky et al. 2009b). Recently, Montoya-Mendoza et al. (2016) reported *E.cornigerum* (voucher CNHE 10217), from *L.synagris* from Santiaguillo Reef, Veracruz (Gulf of Mexico). Examination of that voucher allowed us to confirm the species identity.

##### Specimens deposited.

Six reference specimens in the CNHE (10603).

#### Haliotrema gracilihamus

Taxon classificationAnimaliaMazocraeideaDactylogyridae

Zhukov, 1976

Haliotrema cornigerum Zhukov, 1976: 37–38, fig. 4; Kritsky et al. 2009b: 32–33, figs 79–85 (transferred to Haliotrematoides).

##### Type host.

*Lutjanus apodus*

##### Present study.

*L. griseus*

##### Locality/prevalence, mean abundance and intensity range.

**San Francisco**: 61 fish (mean TL 28.1cm; range 21.6–39) infected of 65 examined (94 %); abundance, 13; intensity of infection, 7–20 worms. **Seyba Playa**: 30 fish (TL 28.5; 23–37) infected of 45 examined (66.6 %); abundance, 8; intensity of infection, 3–8. **Champoton**: 35 fish (TL 28.3; 25.2–37) infected of 37 examined (94.6 %); abundance, 11; intensity of infection, 8–15.

##### Supplementary observations

**(measurements based on ten specimens).** Body 345 (270–400; 10) long; greatest width 75 (60–95; 5). Haptor 67 (60–75; 6) wide. Pharynx 13 wide. MCO 35 (31–40; 9) long. Ventral anchor 51 (46–54; 10) long, base 18 (17–19; 5) wide; dorsal anchor 65 (61–69; 20) long. Ventral bar 31 (30–34; 5) long; dorsal bar 30 (29–31; 5) long.

##### Comments.

This species was originally described as *Haliotremagracilihamus* on *L.apodus* from Campeche Bay (Area Havana) (Zhukov 1976). Later, it was transferred to *Haliotrematoides* as *Hal.gracilihamus* from which it was redescribed based on specimens found on five lutjanids [*L.griseus*, *L.jocu*, *L.apodus*, *L.cyanopterus*, and *Lutjanusaratus* (Günther, 1864)] from the northern Gulf of Mexico (Florida), Mexican Caribbean (off Isla Mujeres and Quintana Roo), Caribbean Sea off Panama, and the Eastern Pacific off Nicaragua (Kritsky et al. 2009b). This species is differentiated from its congeners in having a coiled tube of the MCO with two complete counterclockwise rings and ventral bar with posteromedial shield-like process, and anteromedial flap having two bilateral pockets. Morphometric comparison of the present material with the redescription of this species provided by Kritsky et al. (2009b) did not reveal any differences.

##### Specimens deposited.

Ten reference specimens in the CNHE (10606).

#### Haliotrema heteracantha

Taxon classificationAnimaliaMazocraeideaDactylogyridae

Zhukov, 1976

Figure 2

Haliotrema heteracantha Zhukov, 1976: 36–37, fig. 3; Kritsky et al. 2009b: 42–43 (transferred to Haliotrematoides).

##### Type host.

*Lutjanus synagris*

##### Present study.

*L.synagris* and *L.griseus*

##### Locality/prevalence, mean abundance and intensity range on *L.synagris*.

**San Francisco**: 40 fish (mean TL 28.2 cm; range 20–35.7) infected of 70 examined (57 %); abundance, 5; intensity of infection, 1–16 worms. **Seyba Playa**: 51 fish (TL28.4; 19.2–30.5) infected of 79 examined (64.5%); abundance, 17; intensity of infection, 2–13. **Champoton**: 35 fish (TL 28.2; 24.7–30.5) infected of 75 examined (46.6 %); abundance, 3; intensity of infection, 1–6.

##### Supplementary observations

**(measurements based on six specimens on *L.synagris*).** Body 418 (330–480; 8) long; greatest width 68. Haptor 80 wide. Pharynx 25 (20– 30; 2) wide. MCO 40 (30–45; 6) long. Ventral anchor 40 (38–42; 9) long; dorsal anchor 50 (47–52; 10) long. Hook 12 (3) long.

##### Comments.

This species was originally described as *Haliotremaheteracantha* from *L.synagris* by Zhukov (1976) who also reported it from other five lutjanids [*L.mahogoni*, *L.apodus*, *Ocyuruschrysurus* (Bloch, 1791), *L.analis*, and *L.griseus*] from Bay of Campeche (Area Havana) (Zhukov 1976). Subsequently, Kritsky et al. (2009b) transferred this monogenean species to *Haliotrematoides* by based on original figures of this species made by Zhukov (1976). It has been stated that *Hal.heteracantha* shows a notable similarity with *Hal.guttati* in the Pacific coast off Mazatlán, Sinaloa Mexico based on the comparative morphology of the anchors (i.e. dorsal and ventral anchors with spurs on the inner surfaces of the anchor shafts), bars, and copulatory complex (see Kritsky et al. 2009b).

**Figure 2.**
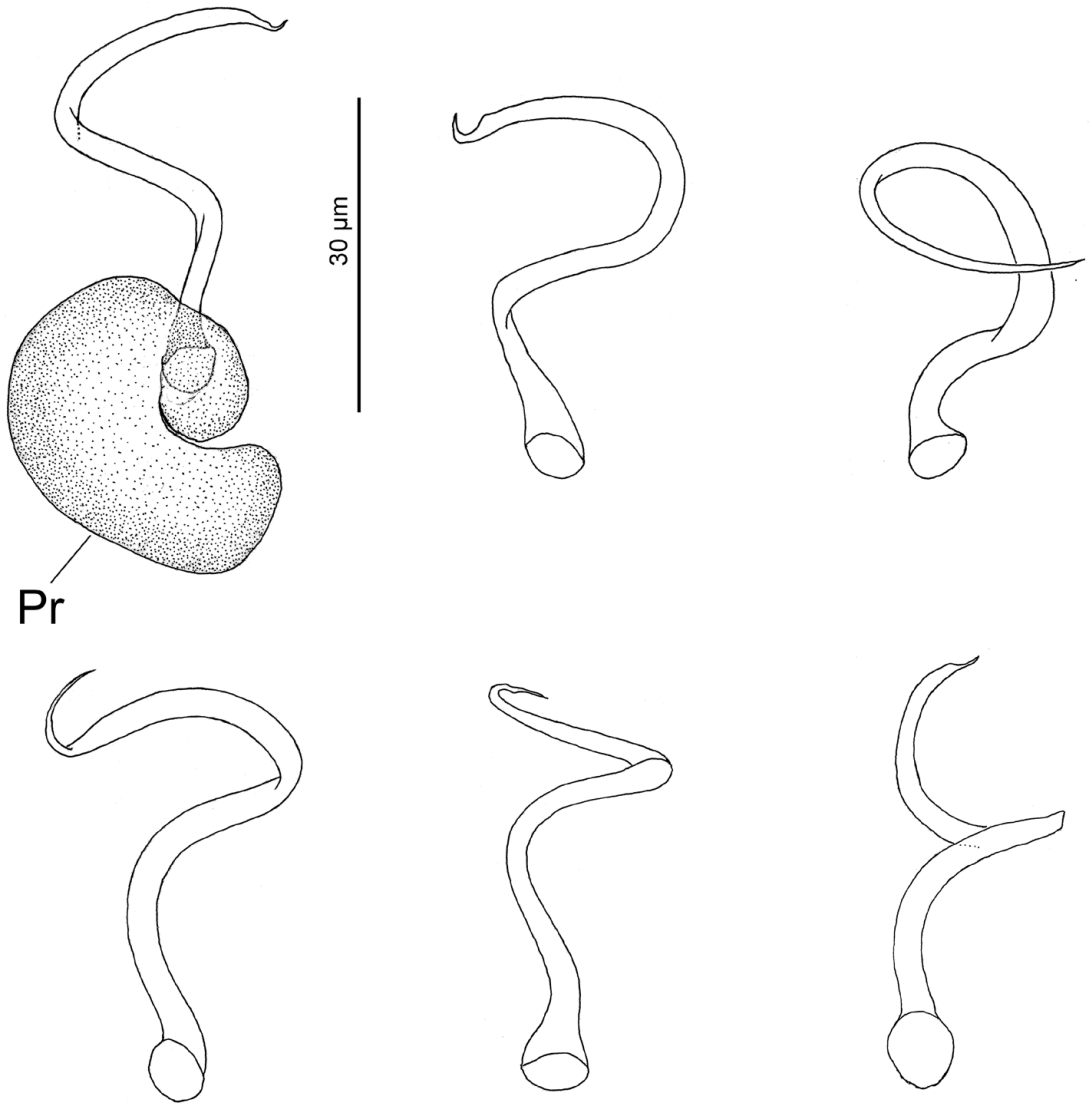
*Haliotrematoidesheteracantha* from *Lutjanussynagris* from Campeche Bank, Mexico: vaginae. Scale bar: 30 µm. Abbreviations: Pr = Prostatic reservoir.

Both monogenean species are currently considered distinct based on the absence of a loop in the shaft of the MCO in *H.heteracantha* (present in *Hal.guttati*). However, examination of present specimens of *H.heteracantha* showed that morphology of the MCO is variable and a loop is present as well in the shaft of the MCO (see Figure 2). Accordingly, it would suggest that *H.guttati* is a junior synonym of *H.heteracantha*. However, the two species have been isolated since formation of the Panamanian Isthmus (~ 3 mya), which theoretically it would support they are distinct species. Sequences of both could probably help in answering the question of conspecificity. Montoya-Mendoza et al. (2016) reported *E.heteracantha* (voucher CNHE 10218) from *L.synagris* from Santiaguillo Reef, Veracruz (Gulf of Mexico). Examination of that voucher allowed us to confirm the species identity.

##### Specimens deposited.

Six reference specimens in the CNHE (10602).

#### Haliotrema longihamus

Taxon classificationAnimaliaMazocraeideaDactylogyridae

Zhukov, 1976

Haliotrema longihamus Zhukov, 1976: 35, fig. 2; Kritsky et al. 2009b: 42 (transferred to Haliotrematoides).

##### Type host.

*Lutjanus synagris*

##### Present study.

*L. synagris*

##### Locality/prevalence, mean abundance and intensity range.

**San Francisco**: 15 fish (mean TL 28.2 cm; range 20–35.7) infected of 70 examined (21.4 %); abundance, 3; intensity of infection, 2–14 worms. **Seyba Playa**: 9 fish (TL 28.4; 19.2–30.5) infected of 79 examined (11.4 %); abundance, 1; intensity of infection, 2–6. **Champoton**: 2 fish (TL 28.2; 24.7–30.5) infected of 75 examined (2.6 %); abundance, 0.04; intensity of infection, 1–2.

##### Supplementary observations

**(measurements based on ten specimens).** Body 475 (390–560; 9) long; greatest width 84 (70–93; 5). Haptor 66 (58–72; 8) wide. Pharynx 25 (24– 30; 7) wide. MCO 48 (40–53; 11) long. Ventral anchor 75 (71–78; 17) long; dorsal anchor 77 (74–80; 17) long. Ventral bar 38 (37–40; 3) long; dorsal bar 39 (37–44; 4) long. Hook 12 (11–12; 10) long.

##### Comments.

*Haliotremalongihamus* Zhukov, 1976 was transferred to *Haliotrematoides* by Kritsky et al. (2009) based on the original figures of the anchor/bar complex and MCO of this species [see Zhukov (1976: fig. 2) and Bosques Rodríguez (2004: fig. 30)]. *Haliotrematoideslongihamus* is characterized by having a longer and convoluted shaft of its MCO and inner blades on the distal portions of the ventral, and dorsal anchor shafts. Currently, *H.longihamus* has been reported on *L.synagris*, *L.mahogoni*, *L.analis*, and *L.griseus* from Bay of Campeche (Area Havana) and Puerto Rico (Zhukov 1976, Bosques Rodríguez 2004, Kritsky et al. 2009). Recently, Montoya-Mendoza et al. (2016) reported *E.longihamus* (voucher CNHE 10219), from *L.synagris* from Santiaguillo Reef, Veracruz (Gulf of Mexico). Examination of that voucher allowed us to confirm the species identity.

##### Specimens deposited.

Ten reference specimens in the CNHE (10599).

#### Haliotrema magnigastrohamus

Taxon classificationAnimaliaMazocraeideaDactylogyridae

Zhukov, 1976

Figure 3

Haliotrema magnigastrohamus Zhukov, 1976: 38, fig. 5; Kritsky et al. 2009b: 40, figs 166–119 (transferred to Haliotrematoides).

##### Type host.

*Lutjanus synagris*

##### Present study.

*L. synagris*

##### Locality/prevalence, mean abundance and intensity range.

**San Francisco**: 65 fish (mean TL 28.2 cm; range 20–35.7) infected of 70 examined 92.8 %); abundance, 9; intensity of infection, 3–13 worms. **Seyba Playa**: 78 fish (TL 28.4; 19.2–30.5) infected of 79 examined (98.7 %); abundance, 17; intensity of infection, 8–28. **Champoton**: 69 fish (TL 28.2; 24.7–30.5) infected of 75 examined (92 %); abundance, 9; intensity of infection, 5–15.

##### Supplementary observations

**(measurements based on ten specimens).** Body 352 (295–382; 7) long; greatest width 66 (50–85; 7). Haptor 64 (52–78; 8) wide. Pharynx 15 (13–19; 3) wide. MCO 28 (25–35; 8) long. Ventral anchor 37 (36–39; 18) long; dorsal anchor 29 (29–31; 17) long. Ventral bar 42 (40–45; 9) long; dorsal bar 17–18 long. Hook 13 (12–13) long.

##### Comments.

**Figure 3.**
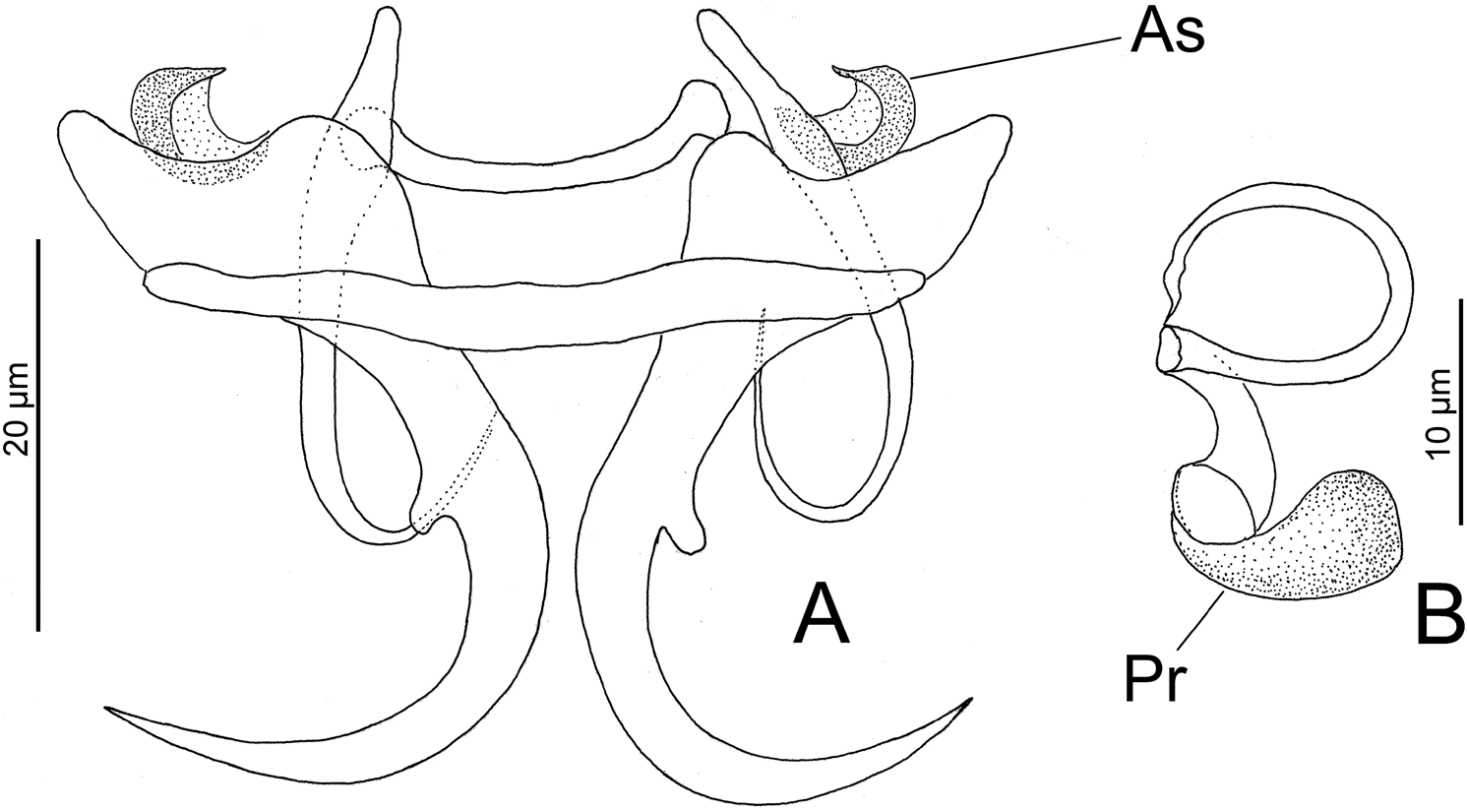
*Haliotrematoidesmagnigastrohamus* from *Lutjanussynagris* from Campeche Bank, Mexico: **A** haptoral armament **B** vagina. Scale bars: 20 µm (**A)**; 10 µm (**B)**. Abbreviations: As = accessory sclerite; Pr = Prostatic reservoir.

This species was originally described by Zhukov (1976) as *Haliotremamagnigastrohamus* from four lutjanid species [*L.synagris*, *L.analis*, *L.mahogoni*, and *O.chrysurus* from the Bay of Campeche (Area Havana)] and later it was transferred to *Haliotrematoides* by Kritsky et al. (2009b) based on specimens of this species found in *L.analis* from Colón, Panama (apparently on the Caribbean Sea off Panama). Present specimens exhibited a C-shaped accessory sclerite (not depicted in Kritsky et al. 2009b) on base of each ventral anchor (see Zhukov 1976; see Figure 3A in the present study). In other features, MCO and dorsal bar (not depicted in Kritsky et al. 2009b) most resembles *Hal.guttati* (García-Vargas, Fajer-Ávila & Lamothe-Argumedo, 2008) Kritsky, Yang & Sun, 2009 from *L.guttatus* from the Mexican Pacific (see figures 107 and 109 in Kritsky et al. 2009b and Figure 3A, B in the present study).

**Figure 4.**
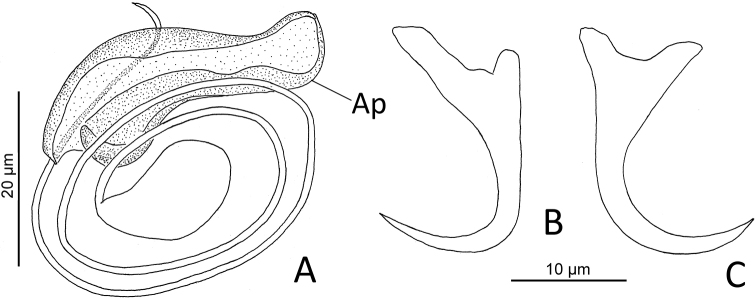
*Euryhaliotrema* sp. (CNHE 10220) from *Lutjanussynagris* from Santiaguillo Reef, Veracruz, México: **A** copulatory complex (dorsal view) **B** ventral anchor **C** dorsal anchor. Scale bar: 20 µm for all figures. Abbreviation: Ap = accessory piece.

Measurements of the present finding fits well with the morphometric of *H.magnigastrohamus* provided by Kritsky et al. (2009b). Montoya-Mendoza et al. (2016) reported *H.magnigastrohamus* on *L.synagris* from Santiaguillo Reef, Veracruz (Gulf of Mexico) and deposited a voucher specimen in the CNHE (accession number 10220). However, examination of that specimen revealed it to be an *Euryhaliotrema* sp. that resembles *E.torquecirrus*. Examination of another voucher specimen labeled as *E.torquecirrus* (CNHE 10223) on *L.synagris* deposited by the same authors revealed it to be same form as that of *Euryhaliotrema* sp. In this latter form, the coil of the MCO comprises 2½ rings (more than four rings in *E.torquecirrus*) and a single accessory piece (accessory piece includes two components in *E.torquecirrus*) (see *Euryhaliotrema* sp. in Figure 4 and *E.torquecirrus* in figure 24 in Kritsty 2012).

##### Specimens deposited.

Ten reference specimens in the CNHE (10600).

#### Haliotrema striatohamus

Taxon classificationAnimaliaMazocraeideaDactylogyridae

Zhukov, 1981

Haliotrema striatohamus Zhukov, 1981: 179, fig. 1; Mendoza-Franco et al. 2009: 1360–1362, figs 1–8 (redescribed and transferred to Haliotrematoides).

##### Type host.

*Haemulonaurolineatum* Cuvier, 1830

##### Present study.

*H. plumieri*

##### Locality/prevalence, mean abundance and intensity range.

**San Francisco**: 80 fish (mean TL 28.8 cm; range 22–34) infected of 90 examined (88.8 %); abundance, 20; intensity of infection, 4–41 worms. **Seyba Playa**: 88 fish (TL 28.9; 21.5–31.3) infected of 90 examined (97.7%); abundance 136; intensity of infection, 30–417. **Champoton**: 90 fish (TL 28.8; 19.3–33.3) infected of 90 examined (100 %); abundance, 91; intensity of infection, 36–166.

##### Supplementary observations

**(measurements based on twelve specimens).** Body 378 (312–450; 12) long; greatest width 78 (62–95; 8). Haptor 68 (55–75; 11) wide. Pharynx 18 (17–20; 5) wide. Copulatory complex 44 (40–55; 9) long. MCO base 20 (20–22; 4) long. Ventral anchor 40 (35–43; 10) long; dorsal anchor 45 (39–47; 12) long. Ventral bar 35 (33–38; 7) long; dorsal bar 42 (40–48; 7) long. Testis 50 (45–55; 3) long, 33 (30–35; 3) wide.

##### Comments.

*Haliotremastriatohamus* Zhukov, 1981 was redescribed and transferred to *Haliotrematoides* Kritsky, Yang & Sun, 2009 by Mendoza-Franco et al. (2009) as *Hal.striatohamus* based on specimens collected from the haemulids, *H.aurolineatum*, *H.plumieri*, and *Haemulonsciurus* (Shaw, 1803) from the southern coast of Quintana Roo, Mexico. This species is characterized in part, in having two accessory sclerites [4 (4–5; 6) long], at the tip of the superficial root of each the ventral anchor.

##### Molecular data.

In the present study, partial molecular sequences (726–746 bp) of the 28S rRNA gene (D1–D3) of *Hal.striatohamus* are provided for the ﬁrst time. These sequences shows that *Hal.striatohamus* collected from Campeche is a sister species of the clade containing *Hal.guttati* and *Haliotrematoidesspinatus* Kritsky & Mendoza-Franco, 2009 (see Figure 1) from *L.guttatus* off Taboga Island (type locality) and Perlas Archipielago in Pacific waters of Panama (Kritsky et al. 2009b).

##### Specimens deposited.

Twelve reference specimens (CNHE 10612).

Two slides, each containing a haptor of a specimen of *Hal.striatohamus* used to amplify its DNA are deposited in the CNHE (10623).

##### Representative DNA sequence.

GenBank accession number MG586869, MG586870.

#### *Hamatopeduncularia* Yamaguti, 1953

##### Hamatopeduncularia bagre

Taxon classificationAnimaliaMazocraeideaDactylogyridae

Hargis, 1955

###### Type host.

*Bagre marinus*

###### Present study.

*B. marinus*

###### Locality/prevalence, mean abundance and intensity range on *B.marinus*.

**San Francisco**: 2 fish (mean TL 42.3 cm; range 38–45.3) infected of 4 examined (50 %); abundance, 1; intensity of infection, 2 worms. **Seyba Playa**: 2 fish (TL 28.3; 27–41.5) infected of 4 examined (50 %); abundance, 0.5; intensity of infection, 1. **Champoton**: 36 fish (TL 28.3; 30–45.2) infected of 43 examined (83. 7%); abundance, 3; intensity of infection, 2–6.

###### Supplementary observations

**(measurements based on eleven specimens on *B.marinus*).** Body 1,463 (1,200–1,850; 10) long; greatest width 217 (175–262; 11). Pharynx 87 (70–112; 5) wide. MCO a coiled tube with 1 counterclockwise ring 70 (58– 97; 10) long, ring 24 (21–30; n=5) diameter. Haptor 324 (262–395; 5) wide. Ventral anchor 61 (68–101; 10) long; base 20 (18–22; 3) wide. Dorsal anchor 247 (225–262; 15) long. Ventral bar 88 (68–101; 7) long. Dorsal bar 146 (125–180; 13) long. Germarium 162 (145–190; 3) long, 66 (62–70; 3) wide. Testis 390 long, 100 wide.

###### Comments.

*Hamatopedunculariabagre* was originally described on *B.marinus* from Alligator Harbor, Franklin County, Florida, USA (Hargis 1955a). Recently, this species was redescribed based on specimens found on another catfish, *Bagrebagre* (Linnaeus, 1766) from several localities in Brazil (Domingues et al. 2016). This monogenean species is characterized mainly by the possession of hooks on haptoral digits, double dorsal bar, and dissimilarity in the size of anchors. Measurements and the morphology of the sclerotized structures of the present specimens do not differ significantly from that figured in the redescription of *H.bagre*.

###### Molecular data.

A 768–770 bp fragment of the 28S rRNA gene (D1–D3) of *H.bagre* on *B.marinus* was obtained in the present study, which represents the ﬁrst molecular data for this monogenean. There are three sequences of individual specimens of *H.bagre* included into the analyses that revealed that this species forms a sister lineage to that containing *N.felis* (see Figure 1).

###### Specimens deposited.

Eleven reference specimens from *B.marinus* in the CNHE (10615).

Three slides, each containing a haptor of a specimen of *H.bagre* used to amplify its DNA are deposited in the CNHE (10627).

###### Representative DNA sequence.

GenBank accession numbers MG586871, MG586872, MG586873.

#### *Neotetraonchus* Bravo-Hollis, 1968

##### Neotetraonchus bravohollisae

Taxon classificationAnimaliaMazocraeideaDactylogyridae

Paperna, 1977

###### Type host.

*Ariopsis felis*

###### Present study.

*A. felis*

###### Supplementary observations

**(measurements based on seven specimens).** Body 890 (762–1,025; 7) long; greatest width 158 (107–200; 7). Pharynx 67–80 wide. MCO 81 (70–90; 7) long. Haptor 128 (105–145) wide. Onchium 39 (32–43; 5) long. Ventral anchor 40 (37–42; n=8) long; dorsal anchor 42 (38–45; 9). Ventral bar 61 (56–65; 4) long; dorsal bar 44 (38–49; 4) long. Hook pair 7, 46 (40–52; 5).

###### Comments.

In *A.felis* a simultaneous infection with *N.felis* was found. Since all worms could not be identified, the data on infection rate relate to *N.bravohollisae* and *N.felis*. *Neotetraonchusbravohollisae* was originally described on *Galeichthysfelis* (Linnaeus) (now *A.felis*), from Dauphin Island, Alabama coast, Gulf of Mexico (Paperna 1977), and posteriorly reported on *Hexanemathichthysassimilis* [now *Ariopsisassimilis* (Günther, 1864)] from Chetumal Bay, Yucatan, Peninsula on the border between Mexico and Belize (Aguirre-Macedo et al. 2007). More recently, *N.bravohollisae* was redescribed based on its type specimens and other specimens collected on *A.felis* in the Gulf of Mexico off the Yucatan, Peninsula Gulf of Mexico (Telchac Puerto and Port of Celestun) (Kritsky et al. 2009a). Measurements and the morphology of the sclerotized structures of the present specimens fit well with those redescribed by these latter authors.

###### Specimens deposited.

Seven reference specimens in the CNHE (10617).

##### Ancyrocephalus felis

Taxon classificationAnimaliaMazocraeideaDactylogyridae

Hargis, 1955

Ancyrocephalus felis Hargis, 1955a: 186–187, figs 28–33; Yamaguti 1963: 66 (transferred to Haliotrema); Paperna 1977: redescribed and transferred to Neotetraonchus; Kritsky et al. 2009a: 9–12, figs 36–44 (redescribed).

###### Type host.

*Ariopsis felis*

###### Present study.

*A. felis*

###### Locality/prevalence, mean abundance and intensity range.

**San Francisco**: 1 fish (mean TL 33.7 cm; range 25–37) infected of 11 examined (9 %); abundance, 0.09; intensity of infection, 1 worm. **Seyba Playa**: 14 fish (TL 32.3; 29–36.5) infected of 27 examined (52%); abundance, 2; intensity of infection, 2–4; **Champoton**: 8 fish (TL 34.2; 27.5–46) infected of 45 examined (17.8%); abundance, 1; intensity of infection, 1–3.

###### Supplementary observations

**(measurements based on four specimens).** Body 2,837 (2,550–3,250; 4) long; greatest width 432 (415–432; 3). MCO 332 (310–368; 4) long. Haptor 197 (155–237; 3) wide. Ventral anchor 41 (40–42; 7) long. Dorsal anchor 35 (35–36; 4) long. Ventral bar 38–48 long. Dorsal bar 35. Hook pair 7, 55 long.

###### Comments.

This species was originally described as *Ancyrocephalusfelis* on *G.felis* (now *A.felis*) from Alligator Harbor, Franklin County, Florida (Hargis 1955a). Yamaguti (1963) transferred this monogenean species to *Haliotrema* as *H.felis* based on the original description and his observations of the type specimens. Paperna (1977) transferred it to *Neotetraonchus* as *N.felis* and added a character within genus, the presence of an accessory piece in the copulatory complex and the onchium (accessory bar) in the haptor (see Kritsky et al. 2009a). Recently, *N.felis* was redescribed based on examination of its type specimen and other new specimens collected on *A.felis* from the Gulf of Mexico off Mississippi and the Yucatan Peninsula (Kritsky et al. 2009a).

Present specimens are clearly conspecific with those of *N.felis* from *A.felis* as redescribed by these latter authors. All these specimens have an elongate tube of the MCO directed posteriorly and reaching level of anterior end of germarium. Currently, *N.felis* has been reported on *A.felis* from Dauphin Island, Alabama coast, Gulf of Mexico (Paperna, 1977); West Ship Island, northern Gulf of Mexico off Mississippi, USA; Gulf of Mexico off Telchac Puerto and Port of Celestun, Yucatan, Mexico (Kritsky et al. 2009a). Present study also provided the ﬁrst molecular data of *N.felis* by amplifying an 772 bp fragment of the 28S rRNA gene (D1–D3). There is one sequence of an individual specimen of *N.felis* included into the analyses that revealed that this species forms a sister lineage to that containing *H.bagre* occurring on other ariids, *B.marinus* and *A.felis* (see Figure 1).

###### Specimens deposited.

Four reference specimens in the CNHE (10616). Another slide containing a haptor of a specimen of *N.felis* used to amplify its DNA is deposited in the CNHE (10801).

###### Representative DNA sequence.

GenBank accession number MG586868.

### Microcotylidae Taschenberg, 1879

#### *Microcotyle* van Beneden & Hesse, 1863

##### Microcotyle archosargi

Taxon classificationAnimaliaMazocraeideaMicrocotylidae

MacCallum, 1913

###### Type host.

*Archosargus probatocephalus*

###### Present study.

*A.rhomboidalis* (new host)

###### Locality/prevalence, mean abundance and intensity range.

**San Francisco**: 17 fish (mean TL 26.9 cm; range 17–23.6) infected of 18 examined (94.4 %); abundance, 4; intensity of infection, 2–6 worms. **Seyba Playa**: 23 fish (TL 28.4; 19.2–30.5) infected of 25 examined (92 %); abundance, 5; intensity of infection, 5–6. **Champoton**: 39 fish (TL 28.2; 24.7–30.5) infected of 45 examined (86.6 %); abundance, 4; intensity of infection, 3–9.

###### Supplementary observations

**(measurements based on nine specimens) in Table 2.**

**Table 2.** Measurements of *Microcotylearchosargi* and *Microcotyletampicensis* (Monogenea) on fishes from the Gulf of Mexico.

Hosts [*Archosargus* (Sparidae) and *Diapterus* spp. (Gerreidae)/ Localities				
Measurements	*M.archosargi* on *A.probatocephalus* from New York, USA (MacCallum 1913)	*M.archosargi* on *A.rhomboidalis* from Campeche Bank (Present study)	*M.archosargi* on *A.probatocephalus* from Florida USA (Kritsky and Bakenhaster 2009)	*M.tampicensis*^†^ on *D.olisthostomus* from Tamaulipas (northern Mexico) (Caballero y Caballero and Bravo-Hollis 1972) Mamaev 1986
Body length	8,000	5,077 (3,775–6,000; n =6)	4,360 (3,950–4,680; n =6)	4,892
Greatest width	800	550 (400–750; n = 8)	413 (329–469; n = 8)	589
Haptor length	–	1,762 (1,050–2,600; n = 3)	–	1,902
Number of clamps	106	127 (121–135; n = 5)	87 (82–90; n = 3)	98
Anterior clamps (Length)	40	44 (36–55; n = 7)	–	45
Posterior clamps	–	33 (28–40; n = 5)	–	29
Anterior clamps (Wide)	80	73 (59–95; n = 7)	–	74
Posterior clamps	–	45 (40–50; n = 6)	53 (49–58; n = 7)	41
Buccal organ length	100	61 (50–70; n = 11)	65 (59–71; n = 8)	74
Wide	–	48 (44–53; n = 7)	90 (76–104; n = 8)	70
Genital atrium length	100	147 (105–180; n = 6)	–	279
Genital atrium width	80 (90–105)^‡^	146 (110–180; n = 7)	149 (132–170; n = 6)	150
Testes number	20-35	16–26	–	13
Length	90	68 (60–80; n = 6)	63 (49–87; n = 12) (diameter)	60
Egg length	170	198 (182–212; n = 6)	–	135

^†^ Measurements from the original description of *Microcotyletampicensis*. ^‡^ Measurements (in round brackets) taken from vouchers.

###### Comments.

Specific placement of current specimens are in agreement with diagnosis provided by MacCallum (1913) who described this species from *A.probatocephalus* obtained from a ﬁsh market (origin unknown) in New York City, USA. Caballero y Caballero and Bravo-Hollis (1972) erected *Paramicrocotyle* to describe *P.tampicensis* and *P.atriobursata* on *Diapterusolisthostomus* (Gerreidae) (now *Diapterusauratus* Ranzani, 1842) from Ciudad Madero, Tamaulipas (Gulf of Mexico) as well as accommodate whithin the genus other sixteen species previously placed in *Microcotyle*, including *M.archosargi*. However, all species of *Paramicrocotyle* were reassigned to *Microcotyle* by Mamaev (1986), who considered *Paramicrocotyle* a junior subjective synonym of *Microcotyle.* Currently, *M.archosargi* (*sensu*Mamaev 1986) has been recorded from sheepshead (as *Archosargusoviceps*) taken at Alligator Harbor, Florida, by Hargis (1956); Iruegas-Buentello (1999) reported it from sheepshead in the Laguna Madre, San Fernando, Tamaulipas, Mexico; and Kritsky and Bakenhaster (2011) provided supplementary observations for *M.archosargi* based on examination of museum specimens and other specimens of this species collected on *A.probatocephalus* from the Indian River Lagoon near Malabar, Brevard County, Florida.

**Figure 5.**
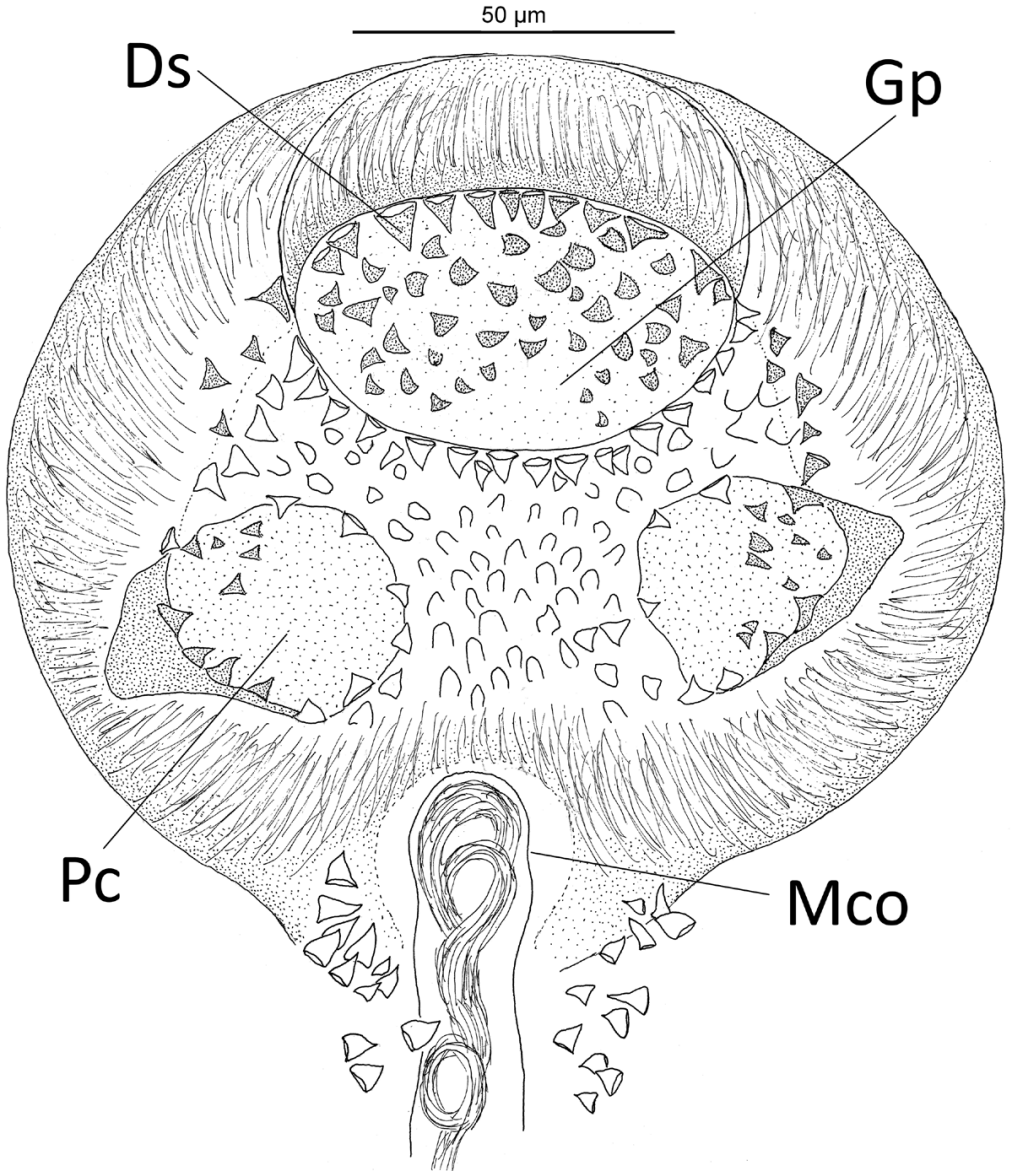
*Microcotylearchosargi* from *Archosargusrhomboidalis* from Campeche Bank, Mexico: genital atrium. Scale bar: 50 µm. Abbreviations: Gp = genital pore; Ds = deeper spines within the body; Pc = posterolateral cavities of the atrium, Mco = male copulatory organ.

These latter authors stated that *M.archosargi* has two bilateral zones of small spines lying slightly posterior to the armed genital atrium, which are close to the ventral surface of the worm, but somewhat deeper within the body than those of the genital atrium. We fully concur in these morphological observations based on examination of present specimens (see Figure 5). Based on examination of five vouchers (CNHE 0323) of *M.tampicensis* (Caballero y Caballero & Bravo-Hollis, 1972), it shows to be extremely similar to the general characteristics of *M.archosargi*, particularly in having morphologically comparable genital atrium (see figures 7–12 in Caballero y Caballero and Bravo-Hollis 1972; figure D in MacCallum 1913; Figure 5 in the present study). The resemblance of both *M.tampicensis* and *M.archosargi* can be explained by the fact that the former was mainly described and/or differentiated of other congeneric species based on the structure and shape of the genital atrium. The two species are presently considered distinct based on the length of the genital atrium, i.e., 279 in *M.tampicensis* vs. 105–180 in *M.archosargi* (see Table 2).

However, the five vouchers of *M.tampicensis* were flattened and/or distorted (i.e., one specimen with distorted genital atrium, two specimens with incomplete haptor and another specimen was fragmented in three parts) due to coverslip pressure, which may have altered the length of the genital atrium. Determination of possible synonymy, therefore, will depend on recollection of the specimens of *M.tampicensis* from *D.olisthostomus* in the Gulf of Mexico for comparison with *M.archosargi*. In other features, present specimens of *M.archosargi* from *A.rhomboidalis* metrically fit within range from those specimens found on *A.probatocephalus* (see Table 2). Differences in the number of testes and clamps, morphologically identical in specimens of *M.archosargi* from different hosts and locations, are considered as intraspecific variation. Montoya-Mendoza et al. (2015) reported *M.archosargi* on *A.probatocephalus* in Veracruz, Mexico, without providing any accession reference number from the CNHE. Then, we could not corroborate finding of these latter authors.

###### Molecular data.

**Figure 6.**
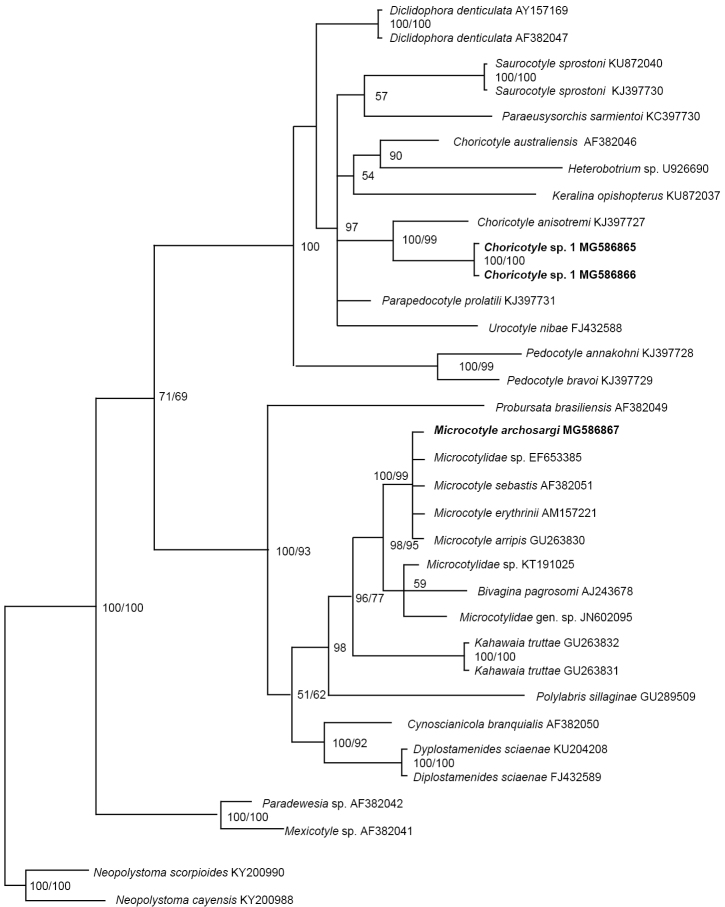
Molecular phylogeny of the Microcotylidae and Diclidophoridae estimated by methods of Bayesian inference (BI) and maximum likelihood (ML) using partial sequences of the 28S rRNA gene (D1–D3). Species newly sequenced for this study are in bold. Species belonging to Polystomatidae were used as outgroups. The species name is followed by the GenBank sequence ID. Posterior probabilities of the BI followed by ML are given above the branches.

This study also provided the ﬁrst molecular data for *M.archosargi* by adding a sequence (638 bp) of an individual specimen into the analyses. This sequence of *M.archosargi* on *A.rhomboidalis* from Campeche supports conspecificity of this monogenean with other microcotylids, i.e., *Microcotylesebastis* Goto, 1894 reported on scorpaeniform hosts (*Sebastodesmaliger* Jordan & Gilbert, 1880, *Sebastodescaurinus* Richardson, 1844 and *Sebastes* sp.) from the UK, Japan, and USA; *Microcotyleerythrini* van Beneden & Hesse, 1863 and *Microcotylearripis* Sandars, 1945 reported on perciformes (*Pagelluserythrini* L.), and *Arripistrutta* (Forster, 1801) (Kaouachi et al. 2010) (see Figure 6).

###### Specimens deposited.

Nine reference specimens in the CNHE (10611). Another slide containing haptor of a specimen of *M.archosargi* used to amplify its DNA is deposited in the CNHE (10626).

###### Representative DNA sequence.

GenBank accession number MG586867.

##### Microcotyle

Taxon classificationAnimaliaMazocraeideaMicrocotylidae

sp.

###### Present study.

*Archosargusrhomboidalis*.

###### Supplementary observations

**(measurements based on three specimens).** Body 3,927 (3,235–4,950; 3) long. Maximum width 493 (310–670; 3) at germarium level. Two oral suckers 67 (55–80; 6) long by 44 (38–49; 6) width. Opisthaptor with eight narrow peduncles. Anterior clamps 34 (32–36; 3) long, 64 (58–72; 5) wide. Genital atrium 161 (155–175; 3) wide, with two bilateral zones of small spines lying posterior to the armed genital atrium and 2 posterolateral cavities. Number of testes 21–22, each subspherical 62 (50–70, 7) long, 67 (62–75; 6) wide. Eggs, 195 (187–200; 3) long, 55–105 wide, each with 2 polar filaments.

###### Comments.

In this host species, *A.rhomboidalis*, a simultaneous infection with *M.archosargi* was found. Since all worms could not be identified, the data on infection rate relate to *Microcotyle* sp. and *M.archosargi*. The present specimens of *Microcotyle* sp. resembles those of *M.archosargi* and *M.atriobursata* [paratype (CNHE 0188) of *Paramicrocotyleatriobursata*] in the general morphology of the genital atrium, 1) two bilateral zones of small spines lying posterior to the armed genital atrium; 2) two posterolateral cavities; 3) genital atrium elliptical, occupying all postbifurcal area; in ventral view, the anterior margin of the atrium is gently curved; posteriorly, the atrium expands into a triangular shape to form an internal cavity surrounded by ventral lips with spines; anterior margin is projected as an operculum on the posterior margin (present in current specimens and *M.atriobursata*) (see figures 1 and 5 in Caballero y Caballero and Bravo-Hollis, 1972; Figure 5 in the present study); 4) number of testes, i.e. 21–22 vs. 20–35 in *M.archosargi* (MacCallum, 1913) and 20–25 in *M.atriobursata*. *Microcotyle* sp. differs from these two microcotylids in the width of the genital atrium, i.e. 155–175 vs. 211–242 in *M.atriobursata* and 80 in *M.archosargi.* Although current specimens are clearly members of the *Microcotyle* they were unsatisfactory to clarify details of internal organs for species identiﬁcation. While intraspecific variation between individuals of *Microcotyle* sp. and *M.archosargi* might represent a single species, new collections of specimens of the former species are necessary for completing formal specific identification of this species.

###### Specimens deposited.

Three reference specimens in the CNHE (10610).

##### Microcotyle incisa

Taxon classificationAnimaliaMazocraeideaMicrocotylidae

Linton, 1910

Microcotyle incisa Linton, 1910: original description; Fujii 1944: 155, figs 9–14 (redescribed and transferred to Microcotyloides).

###### Type host.

*Lutjanus griseus*

###### Present study.

*L. griseus*

###### Locality/prevalence, mean abundance and intensity range.

**San Francisco**: 13 fish (mean TL 28.1cm; range 21.6–39) infected of 65 examined (20 %); abundance, 1; intensity of infection, 1–4 worms. **Seyba Playa**: 24 fish (TL 28.5; 23–37) infected of 45 examined (53 %); abundance, 2; intensity of infection, 2–5; **Champoton**: 10 fish (TL 28.3; 25.2–37) infected of 37 examined (27 %); abundance, 1; intensity of infection, 2–6.

###### Supplementary observations

**(measurements based on eleven specimens).** Body 2,789 (1,100–4,300; 11) long; greatest width 452 (325–700; 9). Oral suckers, each 68 (63–78; 13) long, 41 (30–50; 11) long wide. Clamps, each with 52 (48–60; 8) long, 79 (68–90; 11) wide; posterior clamps 53 (46–55; 6) long, 38 (30–45; 10) wide. Prostatic bulb 79 (70–98; 6) long, 32 (25–35; 3) wide. Testes ovoid, post-ovarian, 21 to 23 in number. Eggs ellipsoidal, each with 223 (200–232; 8) long, 104 (90–123; 8) wide.

###### Comments.

This species was originally assigned to *Microcotyle* based on specimens collected from *L.griseus* in Tortugas, Florida, and the Bermuda Islands, USA (Linton 1910). Later, it was redescribed and transferred to *Microcotyloides* by Fujii (1944) based on specimens collected from the same host and locality. Present specimens from Campeche do not differ significantly from Fujii’s (1944) description. *Microcotyleincisa* is characterized in having a genital atrium without spines, male system with prostatic bulb (70–98 long, 25–35 wide vs. 76–85, 25 in Fujii’s specimens), vaginal pore in right lateral margin of body and eggs attached to one another by the very long, coiled anterior filament around the short posterior filament of the egg in front. *Microcotyloidesincisa* has been reported in the Mexican Pacific on *L.argentiventris*, *L.guttatus*, *Lutjanusjordani* (Gilbert, 1898), and the sciaenid *Umbrinaxanti* Gill, 1862 from Bahia de Chamela, Jalisco; on *Lutjanuscolorado* Jordan & Gilbert, 1882 from Bahia de Banderas in Nayarit; on *L.argentiventris* from la Paz, Baja California; on *L.guttatus* from the coast of Acapulco, Guerrero; on *Cirrhitusrivulatus* Valenciennes, 1846 from the Cabo San Lucas, Baja California Sur, and *Rabiruviainermis* [now *Lutjanusinermis* (Peters, 1869)] from Zihuatanejo; on *L.cyanopterus* from Isla Mujeres (Mexican Caribbean); and on *Neomaensisgriseus* (now *L.griseus*) from Bonnaterre, Florida, EU (Fujii 1944, Mendoza-Garfias and Pérez-Ponce de León 1998).

###### Specimens deposited.

Eleven reference specimens in the CNHE (10605).

### Diclidophoridae Fuhrmann, 1928

#### *Choricotyle* van Beneden & Hesse, 1863

##### Choricotyle

Taxon classificationAnimaliaMazocraeideaDiclidophoridae

sp. 1

Figure 7A, B, F

###### Present study.

*Haemulonplumieri* (new host)

###### Locality/prevalence, abundance and intensity of infection.

**San Francisco**: 53 fish (mean TL 28.8 cm; range 22–34) infected of 90 examined (58.9 %); abundance, 1; intensity of infection, 1–4 worms. **Seyba Playa**: 59 fish (TL 28.9; 21.5–31.3) infected of 90 examined (65.5%); abundance, 3; intensity of infection, 1–6. **Champoton**: 53 fish (TL 28.8; 19.3–33.3) infected of 90 examined (58.9%); abundance, 3; intensity of infection, 2–7.

###### Measurements (based on nine specimens).

**Figure 7.**
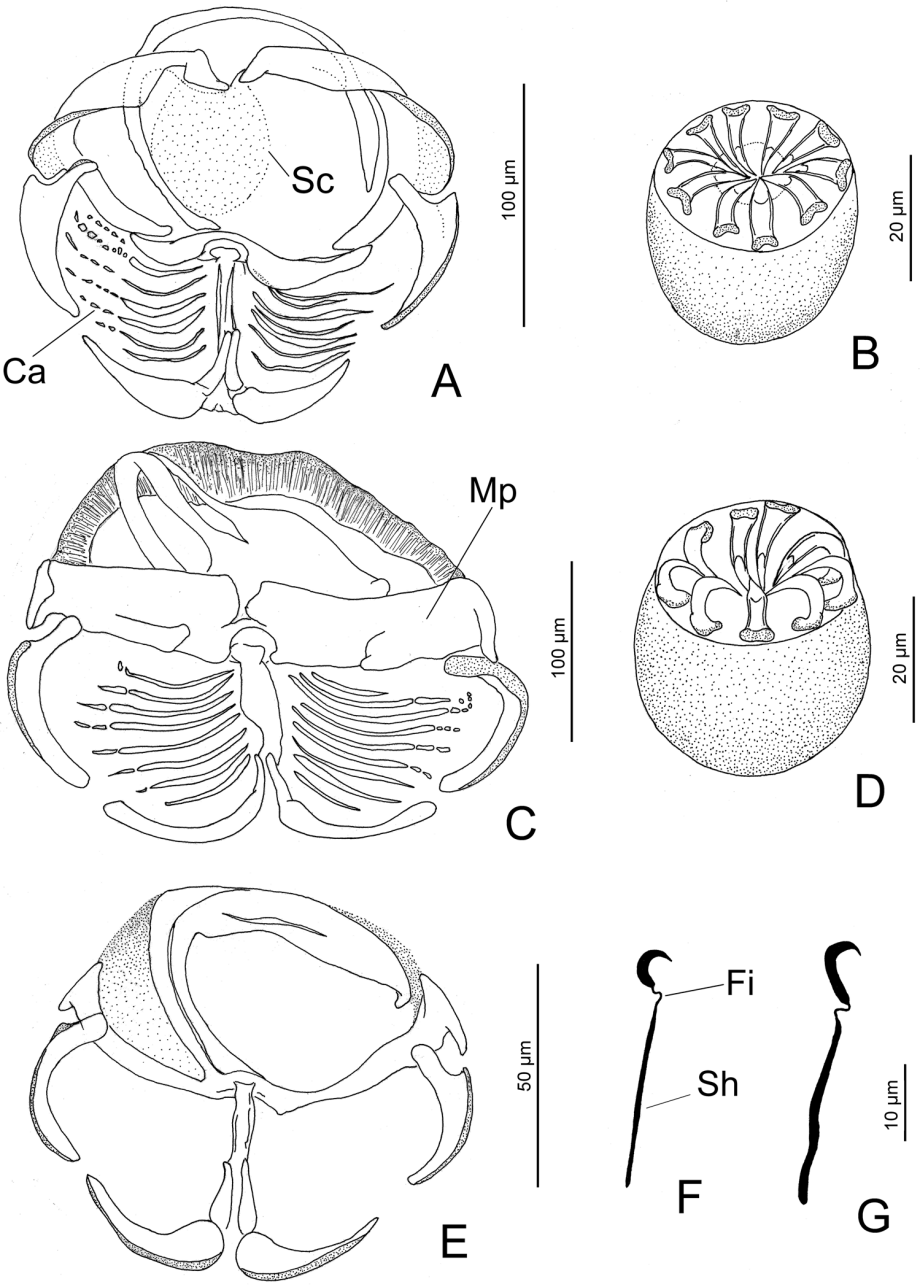
*Choricotyle* spp. from *Haemulonplumieri* from Campeche Bank, Mexico: *Choricotyle* sp. 1. (**A**), clamp (**B**), genital atrium (**F**), hook. *Choricotyle* sp. 2. (**C**), clamp (**D**), genital atrium (**G**), hook. *Choricotyle* sp. 3. (**E**), clamp. Scale bars: 100 µm and 20 µm for all figures, except **E** 50 µm and **F, G** 10 µm. Abbreviations: Ca = concentric arcs; Sc = sucker; Fi = filament; Mp = posterior portion of the medial sclerite; sh = shank.

Body, 2,260 (1,580–3050; 8) long. Maximum width 514 (400–635; 5) at germarium level. Two oral suckers 56 (45–60; 8) long by 40–44 width. Opisthaptor with eight narrow peduncles. Clamps 214 (150–362; 24) long, 191 (130–325; 20) wide, with 6–7 concentric arcs of small skeletal rods in dorsal fields of clamp and an apparent sucker on internal quadrant on clamp (see Ca and Sc in Figure 7A). Terminal lappet on slight posterior protrusion between third and fourth clamp with one pair (at least) of hooks, each 30 (27–33; 3) long, base 7 (6–7; 3) wide, with short filament (see Fi in Figure 7F) connecting shank and base. Genital atrium 39 (30–45; 6) long, 37 (30–45; 8) wide, armed with ten spines in a single concentric row (see Figure 7B). Number of testes 12, each subspherical, 101 (80–145; 4) long, 109 (80–140; 4) wide. Vas deferens, Ootype and Mehli´s gland, seminal receptacle, genito-intestinal canal and oviduct not observed. Eggs, 125–150 long, 65–70 wide, each with two polar filaments.

###### Comments.

Placement of present specimens in *Choricotyle* is based on examination of original descriptions of other species allocated or currently assigned to the genus in Fujii (1944), Hargis (1955b), Kritsky and Bilqees (1973), Oliva (1987), Lamothe-Argumedo et al. (1998), Oliva et al. (2009), and Cohen et al. (2011). While the eight specimens of *Choricotyle* sp. 1 were unsatisfactory to clarify details of internal organs for species identiﬁcation, they appear to represent an undescribed species based on the general morphology of the haptor and genital atrium. *Choricotyle* sp. 1 resembles *C.anisotremi* Oliva, 1987 on *Anisotremusscapularis* (Tschudi, 1846) from Chile; *C.aspinorcha* Hargis, 1955b on *Orthopristischrysopterus* [now *Orthopristischrysoptera* Linnaeus, (1766)] from Beaufort, North Carolina, USA; and *C.hysteroncha* (Fujii, 1944) Sproston, 1946 on *Bathystomastriatum* [now *Haemulonstriatum* Linnaeus, (1758)] (type host), *Brachygenyschrysargyreus* [now *Haemulonchrysargyreus* (Günther, 1859)] and *Haemulonflavolineatum* (Desmarest, 1823) from Tortugas, Florida USA. All these monogeneans share the following features: presence of a sucker on internal quadrant on clamp (present in *Choricotyle* sp. 1 and *C.anisotremi*), relatively similar morphometry of clamps (i.e., 150–362 long × 130–325 vs. 152–219 in diameter in *C.aspinorcha*), number of spines of the genital atrium (10 spines in *Choricotyle* sp. 1 and *C.aspinorcha*), and a lappet with one pair of hooks, each with 27–33 long (one pair, each with 28 long in *C.hysteroncha*). *Choricotyle* sp. 1 differs from these three latter monogenean species by number of testes (12 vs. 90 in *C.anisotremi*, 42–88 in *C.aspinorcha*, and 6–7 in *C.hysteroncha*).

The finding of *Choricotyle* sp. 1 constitutes the second record (the first being that of *Choricotyleleonilavazquezae* Lamothe-Argumedo, Aranda-Cruz & Pérez-Ponce de León, 1998, that occurs on the Pacific coast of Mexico) of a species of *Choricotyle* in Mexico and the first record on *H.plumieri*. In the present study, three species of *Choricotyle* (i.e., *Choricotyle* sp. 1, *Choricotyle* sp. 2 and *Choricotyle* sp. 3) were identified on this latter host species (see below) based on morphological features of the genital atrium, clamps and hooks on terminal lappets (when present), if they actually represent different species since variability in these diclidophorids might exhibit intraspecific differences in the shape or size of these structures above mentioned (see Yang et al. 2007).

###### Molecular data.

The present study also provided the ﬁrst molecular data on species of *Choricotyle* in Mexico; both sequences of *Choricotyle* sp. 1 included into the present analyses revealed that this species forms a sister lineage to that containing *C.anisotremi* (see Figure 6) which occurs on *A.scapularis* (Pomadasyidae) from Chile (Oliva 1987).

###### Specimens deposited.

Nine reference specimens, CNHE (10618).

Other two slides, each containing a haptor of a specimen of *Choricotyle* sp. 1 used to amplify its DNA are deposited in the CNHE (10624 and 10625).

###### Representative DNA sequence.

GenBank accession number MG586865, MG586866.

##### Choricotyle

Taxon classificationAnimaliaMazocraeideaDiclidophoridae

sp. 2

Figure 7C, D, G

###### Present study.

*Haemulonplumieri* (new host)

###### Measurements (based on two specimens [one immature]).

Body, 1,500–2,100 long. Opisthaptor with eight narrow peduncles. Clamps 208 (175–250; 6) long, 235 (200–287; 8) wide with 8–9 concentric arcs of small skeletal rods in dorsal fields of clamp. Terminal lappet at posterior extremity with one pair (at least) of hooks, each 33–35 long, base 8 wide, with short filament (see Figure 4G) connecting shank and base. Genital atrium 45 long, 40–27 wide, armed with nine spines in a single concentric row. Vas deferens, Ootype and Mehli´s gland, seminal receptacle, genito-intestinal canal, oviduct and total number of testes not observed.

###### Comments.

*Choricotyle* sp. 2 has the characteristics and features of *Choricotyle* (i.e. species having four pairs of clamps and genital spines ranging from seven to twelve and exceptionally, from 28 to 30 in *Choricotylerohdei* Cohen, Cardenas, Fernandes & Kohn, 2011). *Choricotyle* sp. 2 appears closest morphologically to *Choricotyle* sp. 1 based on the presence of concentric arcs of small skeletal rods in dorsal fields of clamp and terminal lappet with one pair of hooks of relatively similar size (i.e., 33–35 long vs. 27–33 in *Choricotyle* sp. 1) and having a filament connecting shank and base (see Figure 7F, G). *Choricotyle* sp. 2 differs from *Choricotyle* sp. 1 in the general morphology of clamps (subrectangular vs. rod-shaped posterior portion of the medial sclerite, see Mp in Figure 7C), hooks (robust vs. slender shanks, respectively, see Sh in Figure 7) on the terminal lappet, and number of spines of the genital atrium (9 vs. 10). Only two specimens of *Choricotyle* sp. 2 found on *H.plumieri* that were flattened and unstained with GAP precluded determination of internal anatomy and the consequent speciﬁc assignment of the specimens. A determination may be possible given a more extensive revision of specimens to formally describe this species.

###### Specimens deposited.

Two reference specimens, CNHE (10619).

##### Choricotyle

Taxon classificationAnimaliaMazocraeideaDiclidophoridae

sp. 3

Figure 7E

###### Present study.

*Haemulonplumieri* (new host)

###### Measurements (based on one immature specimen).

Body, 620 long, 200 width. Opisthaptor with eight narrow peduncles. Clamps 196 (157–225; 3) long, 184 (200–287; 4) wide. Buccal organ, each 36–38 long. Vas deferens, Ootype and Mehli´s gland, seminal receptacle, genito-intestinal canal, oviduct and total number of testes not observed.

###### Comments.

*Haemulonplumieri* was revealed to be simultaneously infected with *Choricotyle* sp. 1, *Choricotyle* sp. 2 and *Choricotyle* sp. 3. Because all worms could not be identified, the data on infection rate in *Choricotyle* sp. 1 relate to other two species, *Choricotyle* sp. 2 and *Choricotyle* sp. 3. A single specimen of this latter species collected from *H.plumieri* was immature (less than one mm in total length). Reproductive organs were minimally or undeveloped to determine the specific assignment precluding resolution of the specimen as new or previously described. However, assignment of the current specimen to *Choricotyle* is based on the morphological similarity of its haptoral sclerites to those of species of *Choricotyle* described above on *H.plumieri*.

###### Specimen deposited.

One reference specimen, CNHE (10620).

## Discussion

In this study, we identified 23 gill-infecting monogenean species, assigned to three families (Dactylogyridae, Microcotylidae and Diclidophoridae) and seven genera (*Euryhaliotrema*, *Haliotrematoides*, *Hamatopeduncularia*, *Neotetraonchus*, *Microcotyle*, *Microcotyloides*, and *Choricotyle*), on marine fishes belonging to four families (Lutjanidae, Ariidae, Sparidae, and Haemulidae) from the Campeche Bank (southwest Gulf of Mexico) (see Table 1). *Lutjanusgriseus* in the Campeche Bank represent new host and locality records for *E.fajeravilae* and *E.longibaculum*, and only new locality record for all other dactylogyrids. *Archosargusrhomboidalis* in Campeche represent new host and locality records for *E.amydrum*, *E.dunlapae*, *E.carbuncularium*, *M.archosargi*, and *Microcotyle* sp., *Ariopsisfelis* and *H.plumieri* represent new host species for *H.bagre* and species of *Choricotyle*, respectively.

Most monogeneans found on lutjanids in the present study were originally described from the area Havana (Gulf of Mexico) by Zhukov (1976). However, it is not known if Zhukov obtained lutjanids [i.e., *O.chrysurus*, *L.apodus*, and *L.analis* (all reported as hosts of *H.heteracantha*, *H.gracilihamus*, *E.paracanthi*, *E.fastigatum*, and *H.magnigastrohamus*)] in fish markets or within a commercial fisheries landing site wherein all fishermen might have been working within a radius of the harbor/Havana or if he may have known that some boats were fishing in the Campeche Bank (or the whole Gulf) but landing those Campeche fishes in the port of Havana. In any case, the present survey of monogeneans on lutjanids in the Campeche Bank could represent new locality records as stated in Table 1.

In the Campeche Bank, *E.fajeravilae* on *L.griseus* is reported for the first time; this monogenean species along with *E.fastigatum*, *E.paracanthi*, *Hal.gracilihamus*, and *M.incisa* in the Gulf of Mexico have previously been described and/or reported from the Pacific (Kritsky 2012, Kritsky and Boeger 2002, Kritsky et al. 2009, Mendoza-Garfías and Pérez-Ponce de León 1998). The occurrence of geminate species pairs of Monogenea off North America (as those mentioned above) has been thought to have developed through a vicariant co-evolutionary model when the Panamanian Isthmus divided historical host and parasite distributions into eastern Pacific and western Atlantic populations about 3.2 mya (see Kritsky 2012). However, considering the amount of time that has passed since the closing of the isthmus, and that monogeneans from the two oceans are so close morphologically (i.e., the putative pair represented by *Hal.heteracantha* in the Gulf of Mexico and *Hal.guttati* in the Pacific; see Comments for *Hal.heteracantha*) as to preclude separation is an issue that remains unclear. In fact, some monogenean species ranging on both sides of the isthmus have been provisionally accepted as different species until the putative impact of the Panamanian Isthmus on speciation within this group of parasites is determined (see Kritsky 2012). These putative pairs could suggests that differentiation of morphological features in the Monogenea is a comparatively long process, which in the amphiamerican clades resulted in only slight to insignificant morphological changes developing over the extended period of 3.2 mya and/or speciation is only evident at molecular level (Kritsky 2012). The point is that other monogenean species could have speciated independently on their respective hosts in both sides of the Isthmus.

Molecular data from the present study provides evidence supporting morphological speciation of other monogeneans occurring on both sides of Isthmus. For example, *E.carbuncularium* from *A.rhomboidalis* from Campeche (Gulf of Mexico) appears to be phylogenetically associated with *E.mehen* from *L.guttatus* in the Eastern Pacific. Similarly, *Haliotrematoidesstriatohamus* from *Haemulonplumieri* appears to be a sister species of the clade containing *Haliotrematoidesguttati* and *Haliotrematoidesspinatus*, both from *L.guttatus* in the Pacific waters of Panama (see Figure 4).

In summary, the present study provided six novel sequences of the 28S rRNA gene that advance our understanding of the morphology and host-parasite associations of other monogenean groups. For example, *M.archosargi* from the sparid *A.rhomboidalis* from Campeche clustered with other microcotylids (*M.sebastis*, *M.erythrini* and *M.arripis*) described and/or reported on perciform (Sparidae) and scorpaeniform (Sebastidae) fishes (Figure 6). All these microcotylids exhibit little differentiation at the molecular level despite substantial morphological differentiation on their respective geographically distant host species. Thus, either the 28S rRNA gene is a highly conserved region in these microcotylids or these monogeneans represent same species. Sequences of mitochondrial DNA COI could allow a better phylogenetic resolution of these monogeneans. However, knowledge of potential genes to be amplified in these monogeneans is very poorly known, especially for marine tropical species.

Similarly, in some instances, congeneric and phylogenetically related monogeneans infecting hosts of the same family appear to be phylogenetically closely related based on 28S rRNA gene. For example, *Choricotyle* sp. 1 from the haemulid *H.plumieri* appears to be related to *C.anisotremi* on another haemulid, *A.scapularis* from Chile. Finally, sequences of *H.bagre* on *B.marinus* (present study) (also present on *B.bagre* from Brazil) show that this monogenean is a sister species of *N.felis* on *A.felis* (see Figure 1), with both monogeneans on their respective ariid catfishes occurring from the western Atlantic (i.e. Florida and off Mississippi USA, Gulf of Mexico, Telchac Puerto and Port of Celestun, Yucatan, Mexico and northern Brazil). The relationship observed between these monogeneans is also congruent with that revealed in the phylogeny of their ariids hosts using also molecular data (see Betancur 2009). For example, the clade containing *B.bagre* and *B.marinus* (hosts of *H.bagre*) represents a basal position and genetically distant to that containing *A.felis* (host of *N.felis*) (see Betancur 2009: fig. A). Furthermore, *B.bagre* appears to be a sister species of *B.marinus* (Betancur 2009). *Bagrebagre* and *B.marinus* share the same monogenean species, *H.bagre*, suggesting that this monogenean has coevolved with both ariid hosts since their divergence from a common ancestor or the same monogenean species was able to infect these two closely-related catfishes after they diverged which is not “coevolved” per se; it is simply a lack of host specificity among congeneric hosts.

## Supplementary Material

XML Treatment for Euryhaliotrema

XML Treatment for Euryhaliotrema carbuncularium

XML Treatment for Euryhaliotrema dunlapae

XML Treatment for Euryhaliotrema fajeravilae

XML Treatment for Haliotrema fastigatum

XML Treatment for Haliotrema longibaculum

XML Treatment for Haliotrema paracanthi

XML Treatment for Haliotrema tubocirrus

XML Treatment for Haliotrema cornigerum

XML Treatment for Haliotrema gracilihamus

XML Treatment for Haliotrema heteracantha

XML Treatment for Haliotrema longihamus

XML Treatment for Haliotrema magnigastrohamus

XML Treatment for Haliotrema striatohamus

XML Treatment for Hamatopeduncularia bagre

XML Treatment for Neotetraonchus bravohollisae

XML Treatment for Ancyrocephalus felis

XML Treatment for Microcotyle archosargi

XML Treatment for Microcotyle

XML Treatment for Microcotyle incisa

XML Treatment for Choricotyle

XML Treatment for Choricotyle

XML Treatment for Choricotyle
